# High-fat diet leads to male reproductive dysfunction by disrupting lipid-droplet-mediated organelle crosstalk

**DOI:** 10.1186/s11658-026-00891-2

**Published:** 2026-03-06

**Authors:** Lu Sun, Ao Wang, Yi Zhang, Jinsi Chen, Peng Huang, Kaixuan Zeng, Shuai Huang, Jiayu Huang, Jin Luo, Jiancheng Wang

**Affiliations:** 1 Center of Scientific Research, Department of Traditional Chinese Medicine, The Seventh Affiliated Hospital, Sun Yat-Sen University, ZhenYuan Road 628, Shenzhen, 518107 Guangdong China; 2 Reproductive Medicine Center, Renmin Hospital of Wuhan University, JieFang Road 238, Wuhan, 430060 Hubei China; 3 National-Local Joint Engineering Research Center for Stem Cells and Regenerative Medicine, Zhongshan School of Medicine, Sun Yat-Sen University, Guangzhou, 510080 China; 4 Department of Traditional Chinese Medicine, The Seventh Affiliated Hospital, Sun Yat-Sen University, Shenzhen, 518107 Guangdong China; 5 School of Medicine, Sun Yat-Sen University, Shenzhen, 518107 Guangdong China; 6 Department of Urology, The Sixth Affiliated Hospital, Sun Yat-Sen University, Guangzhou, 510655 Guangdong China

**Keywords:** Endocrine reproductive disorders, Testosterone, High-fat diet, Lipid droplets, Mitochondria-endoplasmic reticulum contacts

## Abstract

**Background:**

The incidence of reproductive system disorders has been steadily rising in recent years. Moreover, with the rising standard of living, the incidence of metabolic diseases also has been gradually increasing. However, the connection and mechanisms linking reproductive and metabolic diseases are poorly defined.

**Methods:**

For organelle connectivity analysis, we analyzed mitochondria–endoplasmic reticulum (ER) contacts (MERCs) gene expression using a published single-cell RNA sequencing data. The link between lipid droplets (LDs) and actin cytoskeleton was analyzed by mass-spectrometry-based proteomics. By flow-cytometry-based cell sorting coupled with transmission electron microscopy, we explored the LD-mediated mitochondria–endoplasmic reticulum contacts.

**Results:**

We found decreased expression of numerous MERC-associated genes, along with a reduction in Leydig cells (LCs), in high-fat diet (HFD) mice. Mechanistically, LDs downregulated the expression of G-actin, leading to the separation of mitochondria from the ER. From a functional perspective, Firsocostat, a lipogenesis enzyme acetyl-CoA carboxylase (ACC) inhibitor, inhibited LD synthesis, which shortened the distance between mitochondria and the ER, improved their functions, and promoted testosterone synthesis. Finally, targeting the LDs offered a promising therapeutic strategy to improve LC function under high-fat conditions, thereby protecting testicular endocrine function.

**Conclusions:**

HFD leads to reproductive dysfunction by disrupting lipid-droplet-mediated Mito–ER contacts.

**Graphical abstract:**

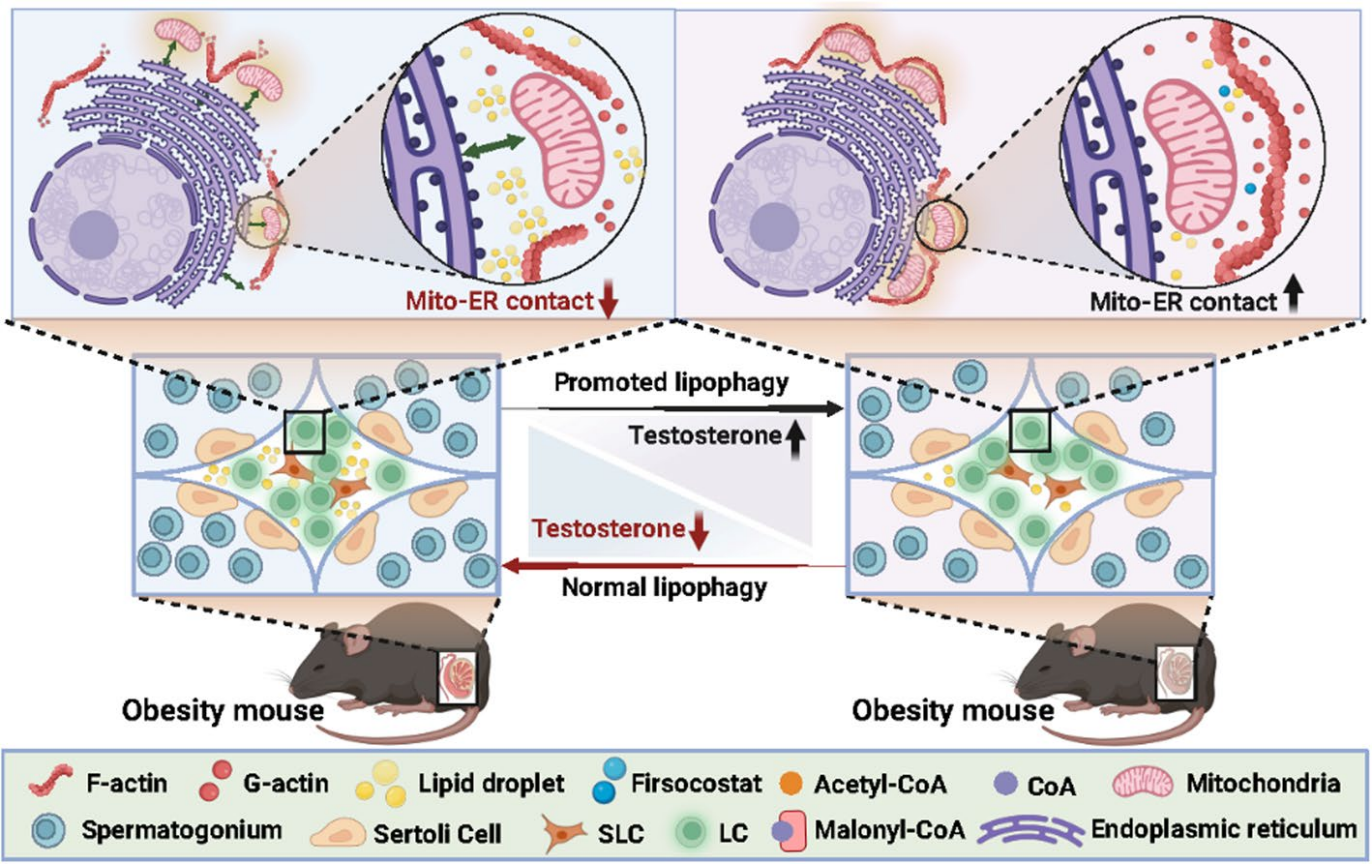

**Supplementary Information:**

The online version contains supplementary material available at 10.1186/s11658-026-00891-2.

## Introduction

In recent years, the global trend toward Westernized diets has led to higher consumption of high-fat diets (HFD) [1], now recognized as a major risk factor for chronic diseases [2, 3] and metabolic syndromes [4–6]. As a highly metabolically active organ, the testis is particularly susceptible to such dietary stress, and increasing evidence links HFD to a decline in male reproductive health [7, 8], acting as a significant environmental trigger for reduced sperm quality [9] and abnormal hormone levels [10]. Given that testicular homeostasis is fundamental to male fertility, it is crucial to understand how HFD induces damage. However, the precise molecular mechanisms by which HFD causes testicular defects, particularly the early subcellular initiating events, remain to be further elucidated.

Leydig cells (LCs) are the primary site of testosterone synthesis in males, providing the necessary androgenic environment for spermatogenesis and the maintenance of male secondary sexual characteristics [11, 12]. Testosterone biosynthesis begins with the side-chain cleavage of cholesterol by the enzyme P450scc within the inner mitochondrial membrane, converting cholesterol into pregnenolone. This initial step is the rate-limiting stage of steroidogenesis and is followed by a series of enzymatic reactions in the endoplasmic reticulum (ER) to produce testosterone [13]. Smooth progression of this synthesis process requires not only normal enzyme activity but also sufficient adenosine triphosphate (ATP) from the mitochondria [14] and ample membrane surface area from the endoplasmic reticulum [15]. Current research indicates that HFD can reduce serum testosterone levels in both experimental animals and humans [16], often owing to HFD-induced inflammation [17], endoplasmic reticulum stress [18, 19], or mitochondrial dysfunction [20]. However, most studies focus on the dysfunction of individual organelles. There is a lack of understanding of how HFD disrupts the physical and functional interactions between mitochondria and the ER at an early stage to interfere with steroid hormone synthesis.

Membrane contact sites are key structures mediating communication and interaction between organelles [21]. Among these, mitochondria-associated membranes (MAMs) are a well-researched example in this field, serving as a highly dynamic functional interface [22]. In this region, mitochondria and the endoplasmic reticulum membrane are separated by a narrow cytoplasmic gap of approximately 10–30 nm, and are connected by specific tethering protein complexes such as the inositol 1,4,5-trisphosphate receptor (IP_3_R)–glucose-regulated protein 75 (Grp75)–voltage-dependent anion channel 1 (VDAC) complex and Mitofusin 2 (MFN2) [23]. This unique ultrastructure provides a physical basis for mitochondrial uptake of calcium ions from the ER [24, 25], thereby influencing mitochondrial energy metabolism, enzymatic reactions, and the transport of lipids such as phospholipids and cholesterol precursors [26]. Additionally, research indicates the cytoskeleton is essential in managing mitochondrial morphology, distribution, and dynamics [27, 28]. Nevertheless, its precise role within MAMs, especially in a testicular physiological environment or under metabolic stress conditions such as high-fat diets, remains an area ripe for exploration.

In our study, we reanalyzed single-cell transcriptome data from the testes of HFD mice from previous research [29] and initially found a significant downregulation of key genes related to the structure and function of MAMs within testicular LCs. This phenomenon suggests that HFD may damage testicular function early by destabilizing interorganelle membrane contacts. Therefore, this study aimed to elucidate how HFD impairs testicular steroidogenesis by specifically targeting the integrity and function of MAMs in Leydig cells. We conducted in-depth experimental validation around this potential molecular mechanism, revealing for the first time the molecular basis of HFD-induced male gonadal damage from the perspective of organelle interactions. This research not only provides new insights into the testicular dysfunction induced by HFD but also lays a potential theoretical foundation for targeted intervention strategies in male reproductive endocrinology.

## Materials and methods

### Animal study

Eight-week-old male C57BL/6 J mice sourced from Top Biotech Biotechnology Co., Ltd. (Shenzhen; IACUC approval no. TOPGM-IACUC-2025–0015) were housed under controlled conditions. The subjects were randomly allocated across three experimental cohorts: control diet (CD), high-fat diet (HFD), CD supplemented with Firsocostat, and HFD supplemented with Firsocostat (*n* = 6 per group). CD animals received standard nourishment, while the HFD cohorts were placed on an 8-week regimen consisting of a diet with 60% fat composition (Research diets, D12492). Body weight was recorded weekly during this period. The CD and HFD plus Firsocostat group received daily intratesticular Firsocostat (HY-16901, MCE) injections at 4 mg/kg body weight for three consecutive days. Subsequently, the mice were euthanized, and serum and testes were collected.

### Leydig cell isolation and fluorescence-activated cell sorting (FACS)

Following isoflurane anesthesia, testicular specimens were procured from CD, HFD, and HFD + Firsocostat mice and immediately submerged in sterile phosphate-buffered saline (PBS) containing 1% penicillin–streptomycin (15140122, Gibco) to remove blood and hair. The tunica albuginea was removed using forceps. The tissue was then minced with iris scissors and enzymatically digested at 37 °C for 15 min using 1 mg/mL collagenase IV (17104-019, Gibco), with shaking every 5 min to ensure thorough digestion. Digestion was terminated by complete medium. The cell suspension was washed twice with PBS. Seminiferous tubules were removed by filtration through a 40-μm filter (431750, Corning). The cell suspension was centrifuged and the pellet was gently resuspended in PBS containing 0.1% bovine serum albumin (BSA) (4240GR100, BioFroxx) and kept on ice for subsequent autofluorescence-based cell sorting.

Autofluorescent cells were subsequently isolated and enriched using an CytExpert SRT (Beckman Coulter) as described [30]. To maximize sorting accuracy while minimizing flow-induced cell damage, specific parameters were implemented. Prior to sorting, the cell suspension was filtered through a 40-μm filter. The sorted cells were collected in a 15-mL centrifuge tube during the sorting process. All flow cytometry data were analyzed using CytExpert SRT (version 1.1) and FlowJo (version 10.8.1).

### Cell culture

TM3 mouse Leydig cells (catalog no. GNM24, Cell Bank of the Chinese Academy of Sciences) were cultured in Dulbecco’s modified Eagle medium (DMEM)/F12 (11320033, Gibco) enriched with 2.5% horse serum (16050122, Gibco) and 5% fetal bovine serum (FBS; BVS500, BioVision Technology) at 37 °C within a 5% CO_2_ incubator. Verification through morphology and function confirmed the feasibility of using TM3 as representative of LCs. To examine the impacts of palmitic acid (PA), a 300 μM solution (KC003, Kunchuang Biotechnology) prepared in 1% BSA was introduced to the cultures for 24 h. The control group received the same volume of 1% BSA. Following this treatment, the cells received an additional 48-h incubation with 70 nM Firsocostat under identical environmental conditions (37 °C, 5% CO_2_).

### Single cell RNA-seq (scRNA-seq) data processing

The scRNA-seq dataset utilized in this investigation was sourced from the Gene Expression Omnibus (GEO) (accession no. GSE239391) [29] and originated from testes of ICR mice exposed to either a chow diet (CD) and high-fat diet (HFD) for 12 weeks. Data preprocessing and analysis were conducted employing Seurat (version 4.1.3). After implementing stringent quality control measures, normalization, nonlinear dimensionality reduction, unsupervised clustering, and cell type identification, numerous downstream investigations were performed. These encompassed visualization of differentially expressed genes (DEGs), evaluations of cellular composition and gene set enrichment analysis (GSEA). Furthermore, gene function was analyzed through Gene Ontology (GO) analysis, and signaling pathways were examined using Kyoto Encyclopedia of Genes and Genomes (KEGG) analysis.

### Semen collection and analysis

Fresh tissue samples from the epididymis were minced into fine segments and transferred into DMEM/F12 containing 0.1% BSA, which had been equilibrated to 37 °C. Following a 15-min incubation period, spermatozoa were permitted to emigrate from the epididymal tissues. In a parallel approach, the caudal epididymal regions were excised, delicately incised using microscissors, and submerged in 0.5 mL of prewarmed medium with 0.5% BSA at 37 °C for 15 min. The sperm was stained with Papanicolaou (Pap) stain (DA0082, Shanghai yuanye Bio-Technology Co., Ltd) to observe its morphology following the manufacturer’s recommended procedures and acquired using DM4B (Leica). Sperm concentration and kinematic parameters were subsequently evaluated with a computer-assisted semen analysis (CASA) platform.

### RNA extraction and real-time quantitative PCR (qPCR)

Half of the testicular tissue specimens or cells were placed into a 2-mL grinding tube containing 1 mL TRIzol reagent (15596026CN, Life Technologies), four 1-mm grinding beads (G0201, Servicebio) were added, followed by lysing using a frozen grinding apparatus (JXFSTPRP-CL-48, Shanghai Jingxin Industrial Development Co., Ltd.). For complementary DNA (cDNA) synthesis, 1 μg of RNA per sample was used for genomic DNA (gDNA) removal and reverse transcription with the HiFiScript All-in-one RT Master Mix (CW3371, CWBIO). Subsequently, qPCR analyses were performed on a CFX96 Real-time System (Bio-Rad) with SYBR qPCR Mix (CW3360, CWBIO) following the manufacturer’s protocols. The specific primer sequences are detailed in Table 1.

**Table 1:** Primers used for qPCR

Gene (mouse)	Sequence (5′–3′)
Star	F: AGGATTGGAAAAGACACGGTC R: CCTCTGCGCTTGGTACAGC
Cyp11a1	AGGTCCTTCAATGAGATCCCTT TCCCTGTAAATGGGGCCATAC
Cyp17a1	F: AGTCAAAGACACCTAATGCCAAG R: ACGTCTGGGGAGAAACGGT
Hsd3β1	F: TGCTGCACAGCCCTCCTA R: TCCATCCAGCCATGGTCAAC
Vdac1	F: GAGTATGGGCTGACGTTTACAG R: GAGCTTCAGTCCACGAGCAAG
Vdac2	F: ATCCCTCCACCCTATGCTGA R: CCAGCCCAAAGCCAAATCCT
Vdac3	F: AAGACCTTCAGCGTTGCCTT R: CCATGCCAAACCCATACCCT
Pdzd8	F: GCTCATTGCTATTGGAGGTGTG R: AGCTTTCTTCCAACTGGCCC
Sig1r	F: CATGGCCATTCGGGACGATA R: CTGGGTGCTGGGTAGAAGAC
Hspa5	F: ACTTGGGGACCACCTATTCCT R: GTTGCCCTGATCGTTGGCTA
Aff4	F: ATGAACCGTGAAGACCGGAAT R: TGCTAGTGACTTTGTATGGCTCA
Atf4	F: CCTGAACAGCGAAGTGTTGG R: TGGAGAACCCATGAGGTTTCAA
Xbp1	F: AGCTTTTACGGGAGAAAACTCAC R: CCTCTGGAACCTCGTCAGGA
Insl3	F: GCTACTGATGCTCCTGGCTC R: GCAGCAGCTCCCGGTC
Inhbb	F: ATCAGCTTTGCAGAGACAGATGG R: TCTCCGTGACCCTGTTCTTG
Gapdh	F: AGGTCGGTGTGAACGGATTTG R: GGGGTCGTTGATGGCAACA

### Protein extraction and western blotting (WB)

Half of the testicular tissue specimens or cells were placed into a 2-mL grinding tube containing radioimmunoprecipitation assay (RIPA) buffer containing a 1 × protease inhibitor cocktail, four 1-mm grinding beads (G0201, Servicebio) were added, followed by lysing using a frozen grinding apparatus (JXFSTPRP-CL-48, Shanghai Jingxin Industrial Development Co., Ltd.). Post-lysis, samples were centrifuged and the supernatant was combined with 5× loading buffer (LT101, Epizyme). The protein was heated at 100 °C for 10 min. Protein concentrations were quantified using a bicinchoninic acid (BCA) assay kit (P0012S, Beyotime). Equivalent protein quantities were separated via sodium dodecyl sulfate (SDS)-polyacrylamide gel electrophoresis (PAGE) on 7.5% (PG111, Epizyme) or 10% (PG112, Epizyme) polyacrylamide gels and transferred to 0.45 μm (IPVH00010, Millipore) or 0.22 μm polyvinylidene fluoride (PVDF) membranes (ISEQ00010, Millipore) on ice. Membranes were blocked and washed twice with 1 mL Tween-20 (GC204002, Servicebio) in Tris-buffered saline (TBS, G0001-2L, Servicebio), and then incubated with primary antibodies overnight at 4 °C. The membranes were exposed to horseradish peroxidase (HRP)-labeled secondary antibodies. Antibody specifications are provided in Table 2. Signal detection was accomplished using an enhanced chemiluminescence substrate (BL520B, Biosharp).

**Table 2:** Antibodies used for WB

Antibody	Source	Identifier	Dilution
F-actin	Abcam	ab130935	1:500
G-actin	Proteintech	11,227–1-AP	1:1000
α-Tubulin	Abcam	ab52866	1:1000
Nestin	Millipore	MAB353	1:500
Bip	Abmart	T55166	1:1000
CHOP	Abmart	T56694	1:1000
Nrf2	Abmart	T55136	1:1000
GAPDH	Proteintech	60,004–1-Ig	1:200,000
Goat anti-rabbit H&L HRP	Abcam	ab205718	1:5000
Goat anti-mouse H&L HRP	Abcam	ab205719	1:5000

### Immunocytochemistry

To investigate the presence of α-Tubulin, Nestin, COX2, PDI, StAR, CYP11A1, and 3β-HSD, the cells underwent a 10-min fixation process at room temperature utilizing 4% paraformaldehyde (PFA; G1101, Servicebio). Following a PBS rinse, the specimens underwent blocking through a 1-h room-temperature treatment with 5% normal serum (ZLI-9056, ORIGEN) and 0.1% Triton X-100 (GC204003, Servicebio) in PBS (PBST). Subsequently, primary antibodies were applied and left to bind at 4 °C overnight. On the following day, the cells received secondary antibody treatment before being counterstained with 4′,6-diamidino-2-phenylindole (DAPI) (BL739, Biosharp). Detailed information of antibodies employed can be found in Table 3.

**Table 3:** Antibodies used for immunostaining

Antibody	Source	Identifier	Dilution
StAR	GeneTex	GTX636800	1:200
CYP11A1	GeneTex	GTX56293	1:200
3β-HSD	Santa Cruz	sc-515120	1:50
SYCP3	Santa Cruz	sc-74569	1:50
CREM	GeneTex	GTX114146	1:100
VDAC1	Proteintech	81,538–1-RR	1: 500
α-Tubulin	Abcam	ab52866	1: 200
Nestin	Millipore	MAB353	1: 200
G-actin	Proteintech	11,227–1-AP	1:300
Tom20	Proteintech	11,802–1-AP	1: 200
PDI	Proteintech	11,245–1-AP	1: 200
COX2	Proteintech	27,308–1-AP	1: 200
Goat anti-rabbit IgG Alexa 488	Invitrogen	A-11008	1:500
Goat anti-mouse IgG Alex 488	Invitrogen	A- 32,723	1:500
Goat anti-mouse IgG Alexa 555	Invitrogen	A-31572	1:500
Goat anti-rabbit IgG Alexa 647	Invitrogen	A- 31,573	1:500
Goat anti-mouse IgG Alex 647	Invitrogen	A-21235	1:500

For examination of 4,4-difluoro-4-bora-3*a*,4*a*-diaza-*s*-indacene (BODIPY) 500/510 C12 (D3823, Invitrogen) and G-actin co-localization, the cells underwent rinsing, fixation, and staining with 5 µM BODIPY dye for 1 h under dark conditions at ambient temperature. After triple washing with PBS, blocking was executed. The samples were then subjected to a G-actin at 4 °C overnight. Following this, the cells received goat-anti rabbit 647 treatment, with nuclei ultimately labeled with DAPI.

Regarding F-actin visualization, cellular samples were processed according to the supplier’s instructions using fluorescein isothiocyanate (FITC)-labeled phalloidin (40735ES75, Yeasen Biotechnology). The procedure began with fixation using 4% PFA, followed by permeabilization with 0.5% PBST. The samples were then incubated with 100 nM FITC-phalloidin for 30 min. After three PBS washes, the slides were mounted with anti-fade mounting medium containing DAPI.

### Immunohistochemistry

For histopathological examination of testicular specimens, the hematoxylin and eosin (H&E) staining protocol was employed. Initially, testicular tissues underwent fixation using a specialized reagent (G1121, Servicebio), after which they were embedded in paraffin and sliced (4 μm). The sections were baked at 65 °C for 4 h to melt the paraffin, followed by two xylene washes to remove the paraffin, and then gradually rehydrated through a descending ethanol series from 100% to 70%. Following a PBS rinse, the samples were stained with hematoxylin and eosin, then dehydrated and mounted under coverslips.

For Oil Red O (ORO) staining, we processed testicular samples by embedding them in OCT (4583, Sakura). Sections measuring 10 μm were prepared using a cryostat (Leica), allowed to air-dry, and briefly rinsed with buffer. The staining procedure involved treating the sections with ORO solution (G1015, Servicebio) for 30 min, followed by a hematoxylin counterstain and final mounting with glycerol gelatin.

All microscopic images were acquired using DM4B (Leica) and subsequently analyzed with ImageJ software.

### Immunofluorescence

To evaluate the immunofluorescence positioning of StAR, 3β-HSD, CYP11A1, SYCP3, and CREM, we processed testicular samples by embedding them in OCT and slicing them into 10-μm sections with a cryostat (Leica). The sections were incubated in a 65 °C oven for 4 h to prevent detachment, followed by three washes with PBS to remove OCT. Then treated with a blocking solution for 1 h. After that, we left the samples to incubate with primary antibodies for a full night at 4 °C, followed by a single-hour fluorescent secondary antibody incubation at room temperature, and finally a DAPI stain. For specific details on the antibodies, see Table 3.

To evaluate the VDAC1 and StAR overlap, we took thin (4 μm) testicular sections, embedded them in paraffin, and baked them at 65 °C for 4 h. After xylene deparaffinization and a descending ethanol rinse, we carried out antigen retrieval and exposed the sections to 3% hydrogen peroxide for 0.5 h. We then treated them with primary antibodies and stored them overnight at 4 °C, followed by a PBS rinse, fluorescent secondary antibody exposure for 1 h, and a final DAPI counterstain. The antibody details are listed in Table 2.

For the BODIPY 500/510 C12 (D3823, Invitrogen) and StAR co-localization study, we air-dried frozen testicular sections, then treated them with a StAR primary antibody and left them to incubate overnight at 4 °C. The next day, we soaked them in a secondary antibody and 5 µM BODIPY dye for 1 h in the dark at room temperature and stained the nuclei with DAPI.

For dihydroethidium (DHE; 309800, Sigma) staining, we air-dried and PBS-washed the frozen sections, then soaked them in 2 µM DHE for 1 h in the dark. Afterward, we washed the sections and mounted them using DAPI-laden anti-fade mounting medium. We captured all images with a DM6B microscope (Leica) and an AX confocal microscope (Nikon), and we analyzed the mean fluorescence intensity (MFI) and colocalization using ImageJ software.

### Transmission electron microscopy (TEM)

Following FACS, the LC sediment was resuspended and preserved in 1 mL of 2.5% glutaraldehyde for 0.5 h while maintaining room-temperature conditions, prior to being refrigerated overnight at 4 °C. Subsequent to this initial fixation procedure, the samples underwent post-fixation treatment with 1% osmium tetroxide for 60 min, followed by an extensive washing protocol before being progressively dehydrated through an ethanol gradient series (commencing with 30%, advancing to 50%, then 70%, and concluding with 95%), with each dehydration interval lasting precisely 5 min. Upon completion of the dehydration process, the specimens were embedded and subjected to polymerization at elevated temperature of 60 °C over a 48-h period. Ultimately, ultrathin sections measuring 80 nm in thickness were prepared and visualized utilizing a JEM-1400 120 kV transmission electron microscope (Jeol Ltd.). Distance measurements were carried out as described previously [31] using Imaris software (Oxford) to add measurement points to the TEM image and calculate the distance between them.

### Testosterone and testicular total cholesterol concentration assay

We utilized a commercially available mouse testosterone enzyme-linked immunosorbent assay (ELISA) kit (AB-B5298, Abmart), following the manufacturer’s recommended procedures. The culture medium of TM3 was collected and centrifuged. The mice were put under anesthesia using isoflurane before whole blood was drawn via cardiac puncture. Following collection, all blood samples underwent centrifugation. Just before running the ELISA, we diluted the serum samples with the kit-provided diluent and loaded them onto the assay plates. Finally, we read the A450 values using a Synergy H1 microplate reader (BioTek), in strict accordance with the kit’s protocol.

To assess total cholesterol concentrations in the testes of both CD and HFD mice, we employed a total cholesterol assay kit (A111-1-1, Nanjing Jiancheng Bioengineering Institute). The testicular tissue was rinsed with ice-cold PBS and then mechanically homogenized in 500 μL of anhydrous ethanol while being kept on ice. For the assay, 2.5 μL of each standard or sample was introduced into the appropriate wells, with blank wells receiving 2.5 μL of ddH_2_O instead. Next, 250 μL of working solution was dispensed into all wells. After ensuring thorough mixing, the microplate was incubated at 37 °C for 10 min, and finally, the A500 values were measured using a Synergy H1 microplate reader (BioTek).

### Measurements of mitochondrial membrane potential and reactive oxygen species (ROS)

The cells were washed by PBS and incubated with 1× JC-1 working solution (C2003S, Beyotime) and 200 nM tetramethylrhodamine, ethyl ester (TMRE, C2001S, Beyotime) at 37 °C for 30 min. Following the staining protocol, the cellular preparations underwent a PBS wash prior to analysis using a CytoFLEX flow cytometer (Beckman Coulter). For evaluating mitochondrial ROS production, the cells were exposed to 1 μM MitoSOX Green (M36005, Invitrogen) and incubated at 37 °C for 30 min. Subsequent to a PBS washing step, the samples were analyzed employing a CytoFLEX flow cytometer (Beckman Coulter). All flow cytometric data were subsequently processed and analyzed using FlowJo software (version 10.8.1).

### Measurements of intracellular ROS

To evaluate intracellular ROS levels in LCs, we treated the cells with 5 μM of the CellROX Deep Red reagent (C10422, Invitrogen), maintaining the cells at 37 °C for a 0.5-h incubation period. Post-incubation, the cells underwent a thorough PBS wash before being analyzed via flow cytometry on a CytoFLEX platform (Beckman Coulter). All collected data were subsequently processed and interpreted utilizing FlowJo software (version 10.8.1).

### Measurements of ATP

We measured ATP concentrations using a commercial kit (S0179, Beyotime) in strict accordance with the manufacturer’s instructions. In short, we first removed the culture medium from the wells of a six-well plate before lysing LCs with 200 µL of lysis buffer per well. Following centrifugation at 12,000*g* for 5 min at 4 °C, we harvested the supernatant for further processing. To create a standard curve, we prepared ATP solutions through serial dilution in lysis buffer, yielding concentrations of 0.01, 0.03, 0.1, 0.3, 1, 3, and 10 µM. For the actual measurements, we mixed 100 µL of the ATP detection working solution with 20 µL of either sample or standard in each well. After a quick homogenization with a pipette and a brief, 2-s incubation period, we captured the luminescence data as relative light units (RLU) employing a Synergy H1 microplate reader (BioTek).

### Measurements of nicotinamide adenine dinucleotide phosphate (NADPH)

The reduced NADPH concentrations were evaluated using a colorimetric NADP^+^/NADPH Assay Kit with WST-8 (S0179, Beyotime). According to the kit’s guidelines, we mixed 200 μL of the NADP^+^/NADPH extract with each million cells to initiate cell lysis. This lysate was then spun down at 12,000*g* at 4 °C for a solid 5-min spin to separate the supernatant, which we set aside for later. We also prepared a suite of NADPH standards—ranging from 0 to 4 μM—by diluting a 1 mM NADPH stock with extraction buffer. We then took 50 μL of each standard or sample, appropriately diluted with the buffer, and placed it in a 96-well plate. Into each well, we added 100 μL of G6PDH working solution, and the plate was left to incubate in the dark at 37 °C for 10 min. Post-incubation, we introduced 10 μL of the chromogenic reagent and thoroughly mixed the contents. The plate was then incubated again, this time in the dark, for 10–20 min. We read the optical density at 450 nm on a BioTek Synergy H1 microplate reader.

### Lipid droplet isolation

We extracted lipid droplets from primary LCs using a commercially available kit (MET-5011, Cell Biolabs) in strict accordance with the manufacturer’s instructions. To begin with, we collected 1.5 × 10^7^ cells and gave them a thorough rinse with PBS. The resulting cell pellet was completely dissolved in 200 µL of Reagent A and left to chill for 10 min in a 2-mL microcentrifuge tube on ice. Following this, we added 800 µL of 1× Reagent B, ensured it was well mixed, and returned the sample to ice for an additional 10 min. To break down the cells, we pushed the mixture five times through a 27-gauge needle (1 inch) fit to a 3-mL syringe. The homogenate received a quick spin in the centrifuge at 100*g* for 5 s. Subsequently, we carefully layered 600 µL of 1× Reagent B on top of the homogenate, drop by drop. Finally, the sample was subjected to centrifugation at 18,000*g* for 3 h while maintaining a temperature of 4 °C. Finally, the top 270 µL was transferred to a fresh microcentrifuge tube for storage at −80 °C.

### Mass spectrometry (MS)

In accordance with well-documented procedures [32], we subjected protein extracts from both the whole LC lysates and the lipid droplets to mass spectrometry. Initially, we dissolved the proteins in 100 mM NaOH and sonicated them at 4 °C. Post-sonication, we neutralized the pH to 7.5 with 200 mM 4-(2-hydroxyethyl)-1-piperazineethanesulfonic acid (HEPES). We then reduced and alkylated the proteins. Each mixture was subsequently diluted six times with ultra-pure water and subjected to an overnight digestion at 37 °C with sequencing-grade trypsin (Worthington Biochemical’s LS02120). Following digestion, the resulting peptides were resuspended and separated using a ThermoFisher Scientific Easy-nLC 1000 system. We acquired mass spectrometry data in the data-dependent acquisition method, focusing on the top ten most abundant precursor ions between 300 and 2000 Da, which were then fragmented for analysis.

### Quantification and statistical analysis

All values are expressed as the mean ± standard error on the mean (SEM), derived from at least three separate experimental runs. Data were analyzed and visualized using GraphPad Prism (version 8.0.2). To analyze differences between two distinct conditions, we employed an unpaired *t*-test. When examining variations across three or more groups, we utilized one-way analysis of variance (ANOVA) supplemented with Tukey’s post hoc test for multiple comparisons. A *p*-value less than 0.05 is considered statistically significant. *p < 0.05; **p < 0.01; ***p < 0.001.

## Results

### HFD damages the male reproductive system

**Fig. 1:**
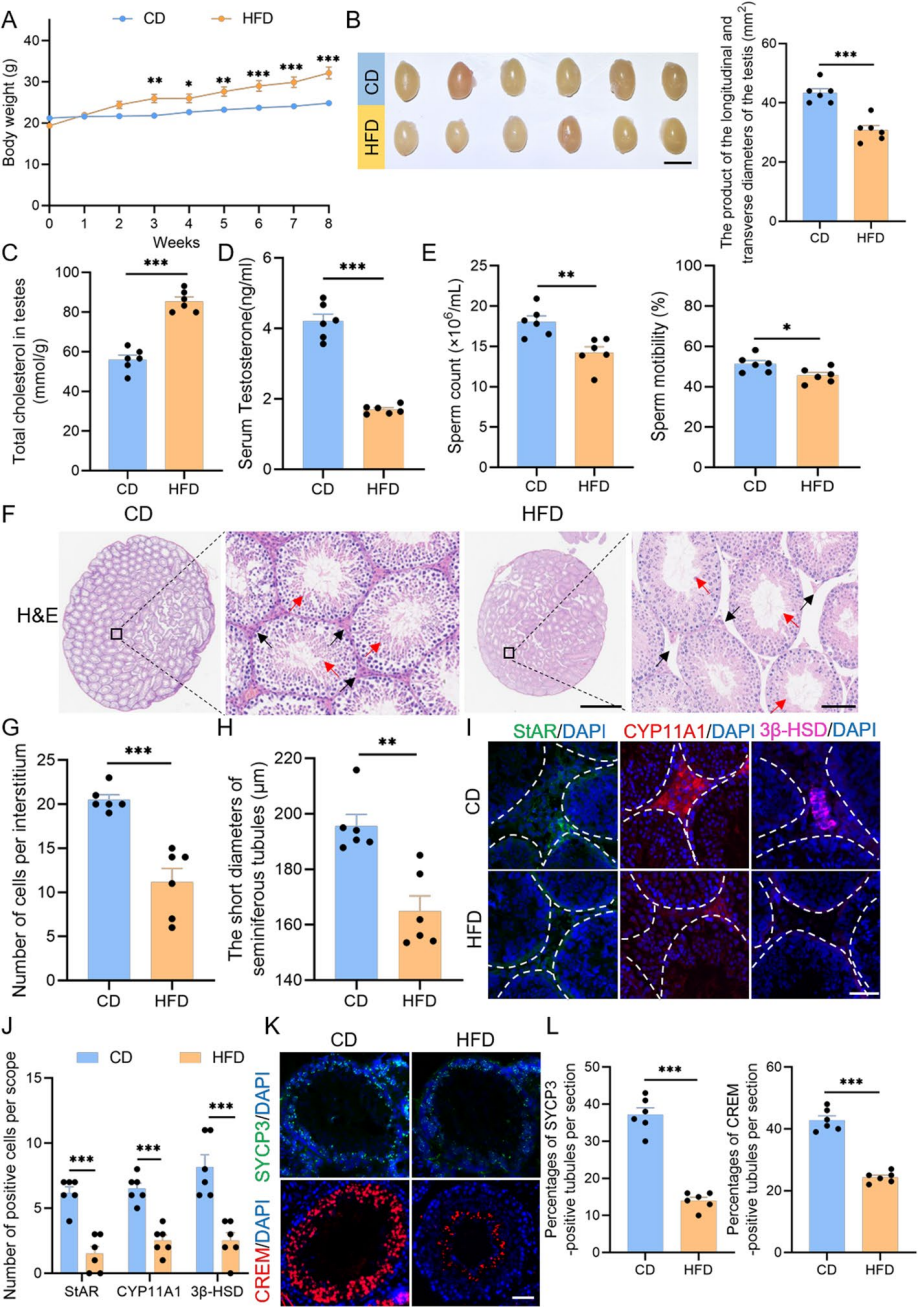
Impaired testosterone synthesis and spermatogenic capacity in HFD mice. **A** Body weight changes in 8-week-old male mice over 8 weeks on a high-fat diet. **B** Sample photographs of testes from CD and HFD-fed mice (left). Scale bar: 0.5 cm. Quantification of the value of testicular length times width (right). **C** Cholesterol content measurements in testicular tissue of CD and HFD mice. **D** Serum testosterone levels in CD and HFD mice. **E** Sperm count and motility in CD and HFD mice (*n* = 6 per group). **F** H&E staining of testicular sections. Black arrows indicate interstitium; red arrows mark seminiferous tubules. Scale bars: 1 mm (overview), 80 μm (magnified views). **G** Quantification of LC numbers per interstitium. **H** Short-axis diameters of seminiferous tubules. **I** Immunofluorescence images of testicular stroma. LCs were identified by StAR/CYP11A1/3β-HSD. Scale bar: 50 μm. **J** Quantification of LC numbers based on immunostaining markers in **(I)**. **K** Immunofluorescence of meiotic spermatocytes (SYCP3, green) and spermatids (CREM, red). Scale bar: 50 μm. **L** Quantification of SYCP3/CREM-positive cells in **(K)**. Each dot represents a mouse. All data are presented as mean ± SEM. Statistical analyses: two-way ANOVA (**A**), unpaired *t* test (**B**–**E**, **G**, **H**, **L**), multiple *t* test (**J**). *p < 0.05; **p < 0.01; ***p < 0.001

To examine the potential impact of a high-fat diet on the male reproductive system, we developed a mouse model fed an HFD. Compared with control mice receiving a normal diet, body weight of HFD mice increased significantly with the duration of high-fat feeding (Fig. 1A), while testis volume and weight were markedly reduced (Fig. 1B, Supplementary Fig. S1A). At the molecular level, lipid homeostasis was clearly disrupted in the testes of HFD mice. Total cholesterol levels were significantly elevated in the HFD testes (Fig. 1C), and ORO staining indicated lipid droplet deposition in the interstitial tissue, rather than in the seminiferous tubules (Supplementary Fig. S1B). Concomitant with these lipid metabolic abnormalities, ROS levels in testicular tissue were also significantly increased (Supplementary Fig. S1C, D). Moreover, serum testosterone levels were significantly decreased in HFD mice (Fig. 1D). Further qPCR analysis showed that the expression of key rate-limiting enzymes in testosterone biosynthesis—Star, Cyp11a1, and Cyp17a1—was significantly downregulated (Supplementary Fig. S1E). As testosterone declined, spermatogenic function in HFD mice was substantially impaired, evidenced by a significant reduction in sperm count and motility (Fig. 1E, Supplementary Fig. S1F). Histological analysis revealed pronounced pathological changes in testicular architecture caused by the HFD (Fig. 1F), characterized by a reduced number of interstitial cells (Fig. 1G) and narrowed seminiferous tubule short diameters (Fig. 1H). Besides, immunohistochemical analysis showed downregulation of steroidogenic enzymes StAR, CYP11A1, and 3β-HSD (Fig. 1I, J) and a lower proportion of spermatogonia in HFD testes (Fig. 1K, L). Furthermore, the expression of key functional markers—Insl3 for LCs and Inhibin B (Inhbb) for Sertoli cells—was also reduced (Supplementary Fig. S1G). These findings indicate that HFD not only damages testicular structure in mice but also markedly disrupts endocrine regulation and spermatogenesis, thereby broadly impairing male reproductive health.

### High-fat diet impaired endoplasmic reticulum–mitochondria contact in mouse LCs

**Fig. 2:**
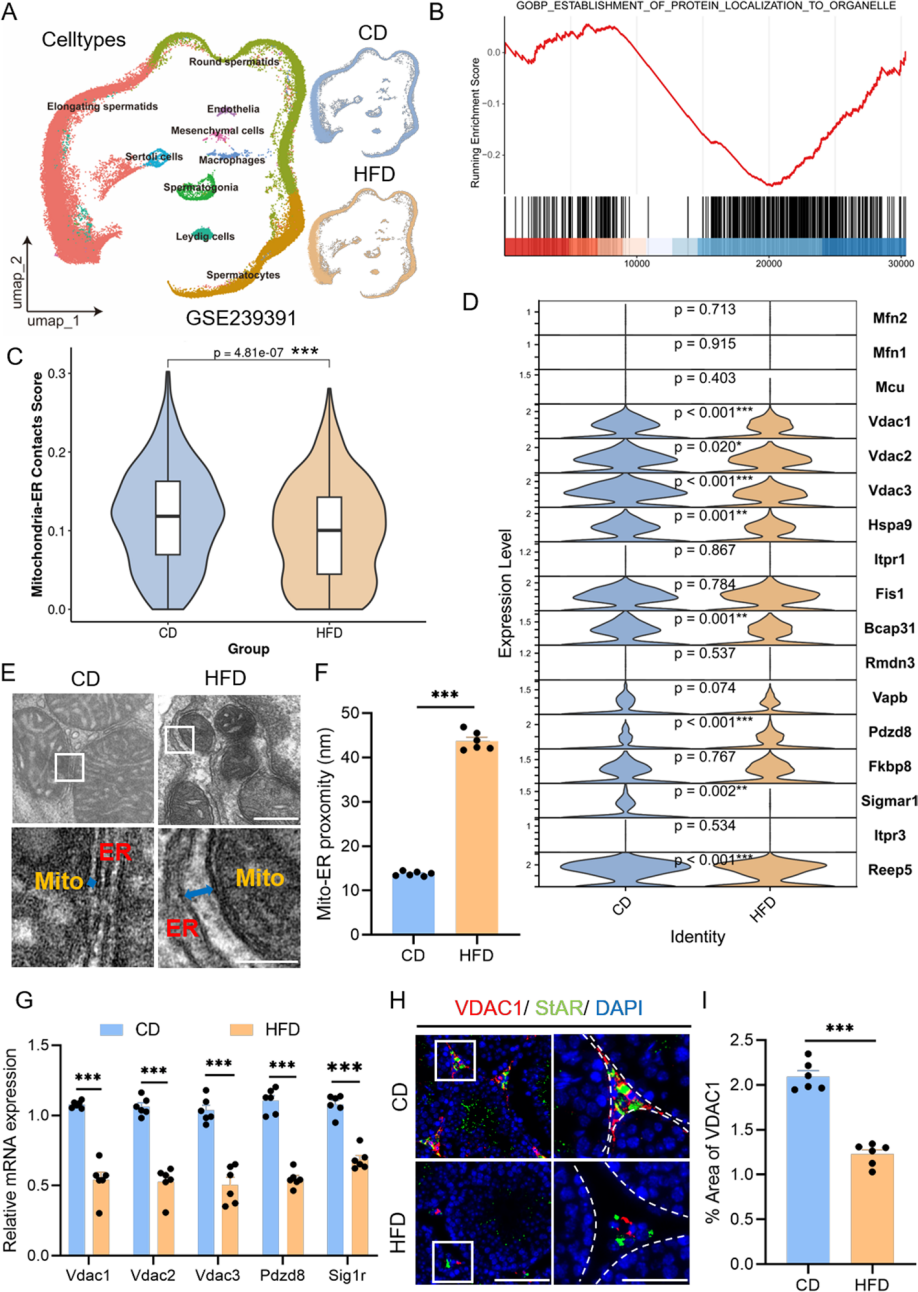
LCs establish fewer MERCs under high-fat conditions. **A** Cell clusters identified from the single-cell RNA-seq data by Cao. et al. [29] in the GSE239391. CD: a chow diet group. **B** GSEA of hallmark gene sets showing significant enrichment of the “response to protein localization to organelle” pathway. **C** AddModuleScore analysis evaluating MERC activity in CD and HFD mice. **D** Violin plots displaying expression levels of MERC-related genes in LCs from CD and HFD mice. **E** Representative TEM images of LCs from CD and HFD testes. Scale bars: 200 nm (overview), 100 nm (enlarged). **F** Quantification of mitochondrial-ER proximity based on (**E**) (*n* = 6 mitochondria across three fields per condition). **G** qPCR analysis of MERC gene expression of testes from CD and HFD mice (*n* = 6 mice per group). **H** Representative immunofluorescence images of VDAC1 (red) and StAR (green) in CD and HFD testes. Scale bars: 100 μm (overview), 50 μm (enlarged). **I** Quantification of VDAC1-positive area from (**H)** (*n* = 6 mice per group). Data are presented as mean ± SEM. Data were analyzed using Wilcoxon rank-sum test (**D**), unpaired *t* test (**F**, **I**), Multiple *t* test (**G**). *p < 0.05; **p < 0.01; ***p < 0.001

To explore the molecular mechanisms by which a high-fat diet affects the testes, we reanalyzed a single-cell transcriptome dataset from the GEO database (Fig. 2A, GSE239391) [29]. This dataset includes testes samples from three CD mice and three HFD mice, with nine cell types identified on the basis of specific markers (Supplementary Fig. S2A). Various cell populations in the testes of HFD mice showed a reduction in number (Supplementary Fig. S2B). To further elucidate the molecular basis of decreased testosterone levels in HFD mice, we isolated LCs—the primary cells responsible for testosterone synthesis—and performed reclustering and differential expression analysis. The results showed that, compared with CD, LCs from HFD mice exhibited 961 significantly upregulated and 1133 significantly downregulated genes, while genes related to steroid biosynthesis were markedly suppressed (Supplementary Fig. S2C, D). GSEA indicated that pathways related to “establishment of protein localization to organelle” were notably downregulated in HFD mice (Fig. 2B), suggesting that HFD may impair interorganelle interactions within LCs. Mitochondria and the ER play crucial roles in energy supply and enzymatic reactions during testosterone biosynthesis, and their structural contact and functional coupling are critical for steroid production. To investigate whether HFD affects this contact, we compared the area under the curve (AUC) scores of gene sets associated with mitochondrial–ER contact in LCs from CD and HFD groups, finding a significant decrease in the HFD group (Fig. 2C). Further analysis of the gene set revealed that key genes such as Vdac1, Vdac2, Vdac3, Hspa9, Bcap31, Sigmar1, and Reep5 were markedly downregulated in HFD mice (Fig. 2D), suggesting that HFD may impair the structural contact between mitochondria and the ER in LCs. To validate the single‑cell findings, we isolated LCs from both groups by flow cytometry for further analysis (Supplementary Fig. S2E). We defined an “effective contact” between mitochondria and the ER as a distance of less than 100 nm [33]. TEM revealed a significant increase in the distance of Mito–ER in LCs from HFD mice (Fig. 2E, F). Quantitative PCR confirmed the gene expression differences observed in the single‑cell analysis (Fig. 2G). Additionally, immunofluorescence staining showed that Vdac1, an outer mitochondrial membrane ion channel protein, was markedly reduced in LCs of HFD mice (Fig. 2H, I). In summary, a high‑fat diet significantly impairs the structural contacts and functional interactions between Mito–ER in LCs, which may underlie the reduction in testosterone synthesis.

### High fat impairs mitochondrial–ER contacts by disrupting actin filaments

**Fig. 3:**
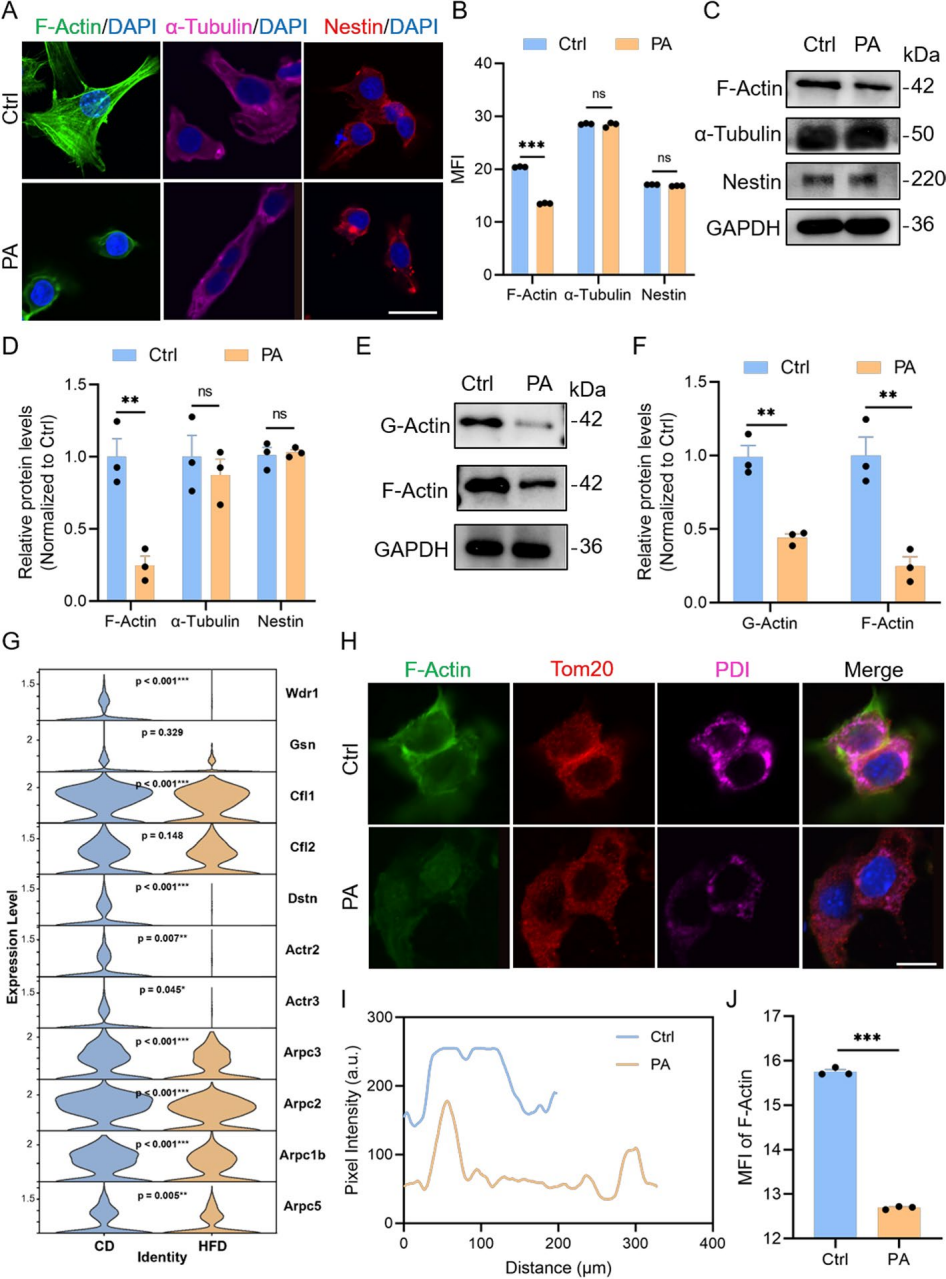
High lipid levels reduce F-actin in LCs, leading to fewer MERCs. **A** Representative immunofluorescence images showing F-actin (green), α-tubulin (magenta), and Nestin (red) in Ctrl and PA-treated TM3 cells. Scale bar: 25 µm. **B** Quantitative analysis of the mean fluorescence intensity for the three cytoskeletal components shown in (**A**). **C**, **D** WB analysis of F-actin, α-tubulin, and Nestin expression in Ctrl and PA-treated TM3 cells. **E**, **F** WB analysis of G-actin and F-actin levels in Ctrl and PA-treated TM3 cells. **G** Violin plots illustrating the expression levels of actin dynamics-related genes in LCs from CD and HFD mice. The full names of the genes in the figure are listed in the abbreviations. **H** Representative immunofluorescence images of F-actin (green), Tom20 (mitochondria, red), and PDI (ER, magenta) in Ctrl and PA-treated TM3 cells. Scale bar: 25 µm. **I** Colocalization analysis of Tom20 and PDI signals from the images in (**H**). **J** Quantitative analysis of the mean fluorescence intensity of F-actin shown in (**H)**. Data are presented as mean ± SEM (*n* = 3 biological replicates per group). Statistical analyses were performed using multiple *t* test (**B**, **D**, **F**), unpaired *t* test (**J**), Wilcoxon rank-sum test (**G**). *p < 0.05; **p < 0.01; ***p < 0.001; ns, *p* ≥ 0.05

To model the effects of a high‑fat environment on LCs at the cellular level, TM3 cells were treated with PA. PA exposure markedly suppressed expression of key steroidogenic genes (Star, Cyp11a1, Cyp17a1, and Hsd3b1) (Supplementary Fig. S3A), consistent with our in vivo data and indicating that a lipid‑rich environment directly impairs steroid biosynthesis in LCs. The cytoskeleton plays a central role in interorganelle communication [31]. We therefore compared cytoskeletal components under different treatments. Under high‑fat conditions, F‑actin distribution in LCs became uneven (Fig. 3A, B) and total F‑actin levels were significantly reduced (Fig. 3C, D), whereas α‑tubulin (microtubules) and Nestin (intermediate filaments) were largely unaffected. G‑actin (monomeric actin) was also significantly decreased after PA treatment (Fig. 3E, F), suggesting that the balance between actin monomer and polymer forms is disturbed by lipid overload. Meanwhile, analysis of prior single‑cell transcriptomic data showed broad downregulation of genes involved in actin dynamics in LCs (Fig. 3G), supporting these observations. Immunofluorescence confirmed that loss of F‑actin was associated with increased mitochondria–ER contacts (MERCs) (Fig. 3H-J). We then assessed mitochondrial function and ER stress in LCs under high‑fat conditions. Abnormal MERCs were accompanied by exacerbated mitochondrial dysfunction and ER stress: mitochondrial membrane potential decreased (Supplementary Fig. S3B, C); total NADPH and ATP levels were significantly reduced (Supplementary Fig. S3D, E); and mitochondrial superoxide production increased markedly (Supplementary Fig. S3F, G). TEM revealed an increase in intracellular lipid droplets (Supplementary Fig. S3H), along with loss and disorganization of cristae and matrix condensation in mitochondria of PA‑treated cells (Supplementary Fig. S3I). Besides, western blotting showed PA treatment activated the antioxidant regulator Nrf2 and upregulated ER stress marker Bip and pro‑apoptotic CHOP (Supplementary Fig. S3J, K). Furthermore, the three core ER stress signaling axes (Hspa5/Atf4, Xbp1, and Atf6 pathways) were significantly induced (Supplementary Fig. S3L). Together, these results indicate that actin cytoskeleton disruption under high‑fat conditions induces abnormal mitochondria–ER contacts, leading to mitochondrial dysfunction and heightened ER stress, which ultimately impairs testosterone biosynthesis.

### Lipid droplets modulate actin structure and function by regulating G‑actin

**Fig. 4:**
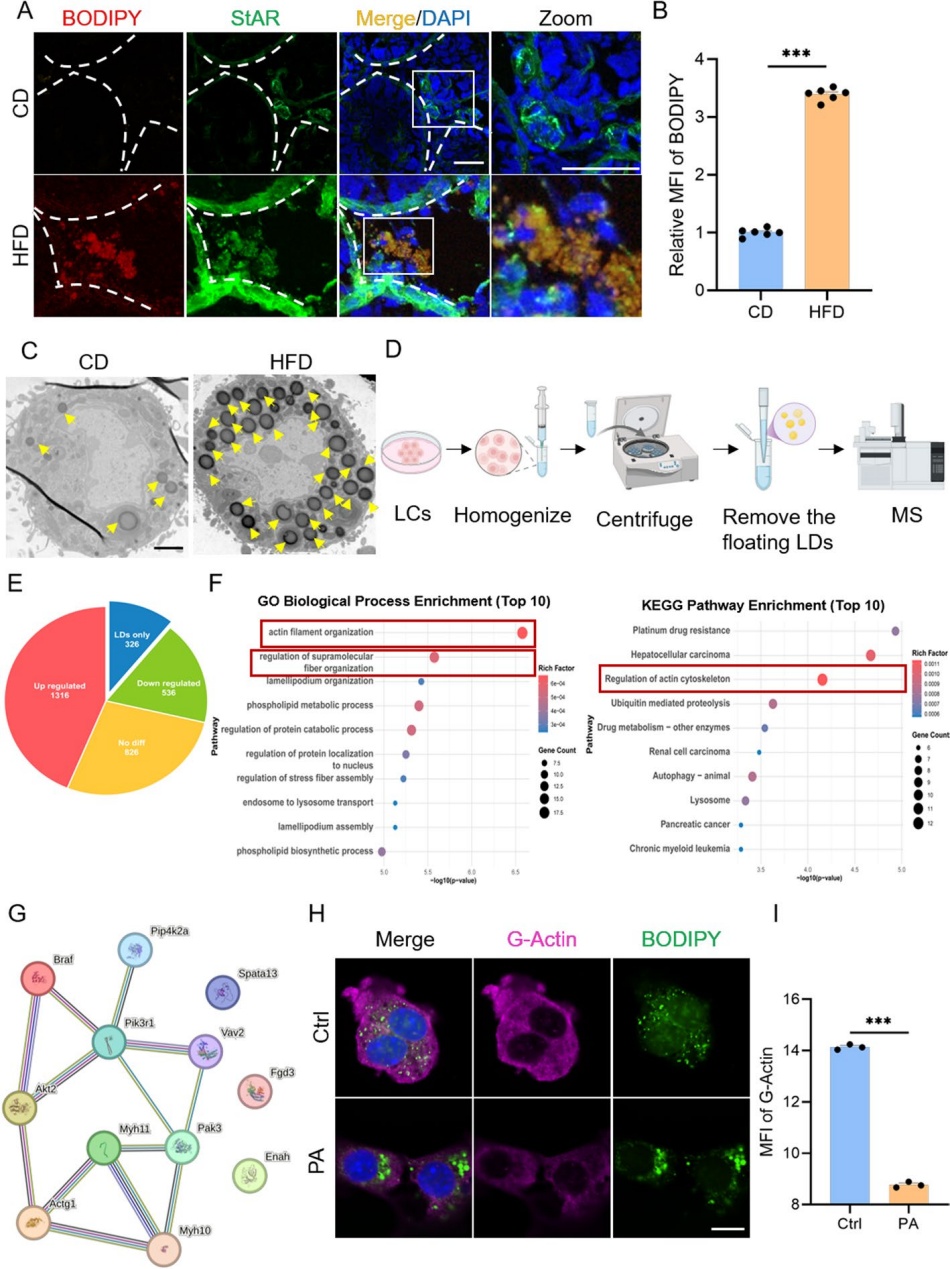
Lipid droplets lead to a reduction in G-actin. **A** Representative immunofluorescence images of BODIPY (red) and StAR (green) in testicular sections from CD and HFD mice. Nuclei were counterstained with DAPI (blue). Scale bars: 50 μm. **B** Quantification of mean BODIPY fluorescence intensity from (**A**) (*n* = 6 mice per group). **C** TEM micrographs showing LDs in CD and HFD LCs, with LDs indicated by yellow arrows. Scale bar: 2 μm. **D** Workflow for isolating LDs from CD and HFD LCs for MS analysis. **E** Protein distribution profiles in whole cells and LD fractions. **F** Top 10 significantly enriched GO biological processes (left) and KEGG pathways (right) associated with LD proteins. **G** Protein–protein interaction (PPI) network of proteins involved in the regulation of the actin cytoskeleton pathway. The full names of the proteins in the figure are listed in the abbreviations. **H** Immunofluorescence staining of G-Actin (magenta) and BODIPY (green) in Ctrl and PA-treated TM3 cells. Nuclei were stained with DAPI (blue). Scale bar: 10 μm. **I** Quantification of mean G-Actin fluorescence intensity from (**H**) (*n* = 3 biological replicates per group). All data are expressed as mean ± SEM. Statistical comparisons were performed using an unpaired *t* test (**B**, **I**). ***p < 0.001

Lipid droplets (LDs) are key organelles for maintaining intracellular lipid homeostasis. In this study, we observed extensive LD accumulation in the testicular interstitium of HFD mice (Fig. 4A, B). Flow cytometric sorted LCs revealed a significantly higher number of intracellular LDs in the HFD group compared with controls (Fig. 4C). Although LDs are known to interact with multiple organelles [34], their role in regulating other cellular structures in LCs remains unclear. To address this, we performed LD-associated proteomics on sorted mouse LCs (Fig. 4D) and identified 326 proteins specifically associated with LDs (LD-only proteins) (Fig. 4E). GO enrichment analysis showed these proteins are primarily involved in “actin filament organization” and “regulation of supramolecular fiber organization”; KEGG pathway analysis further indicated significant enrichment in the “regulation of actin cytoskeleton” pathway (Fig. 4F). These results suggest that LDs may participate in dynamic regulation of the actin cytoskeleton in LCs. Figure 4G displays the proteins involved in the “regulation of actin cytoskeleton” pathway and their interaction network. To validate these findings, we measured G-actin expression under high-fat conditions and found that G-actin levels were markedly decreased in association with LD accumulation (Fig. 4H, I). This result supports the notion that LD accumulation in a lipid-rich environment may reduce monomeric G-actin, impair F-actin polymerization, and consequently disrupt MERC architecture.

### Firsocostat ameliorates HFD-induced MERC disruption and testosterone synthesis defects in LCs

**Fig. 5:**
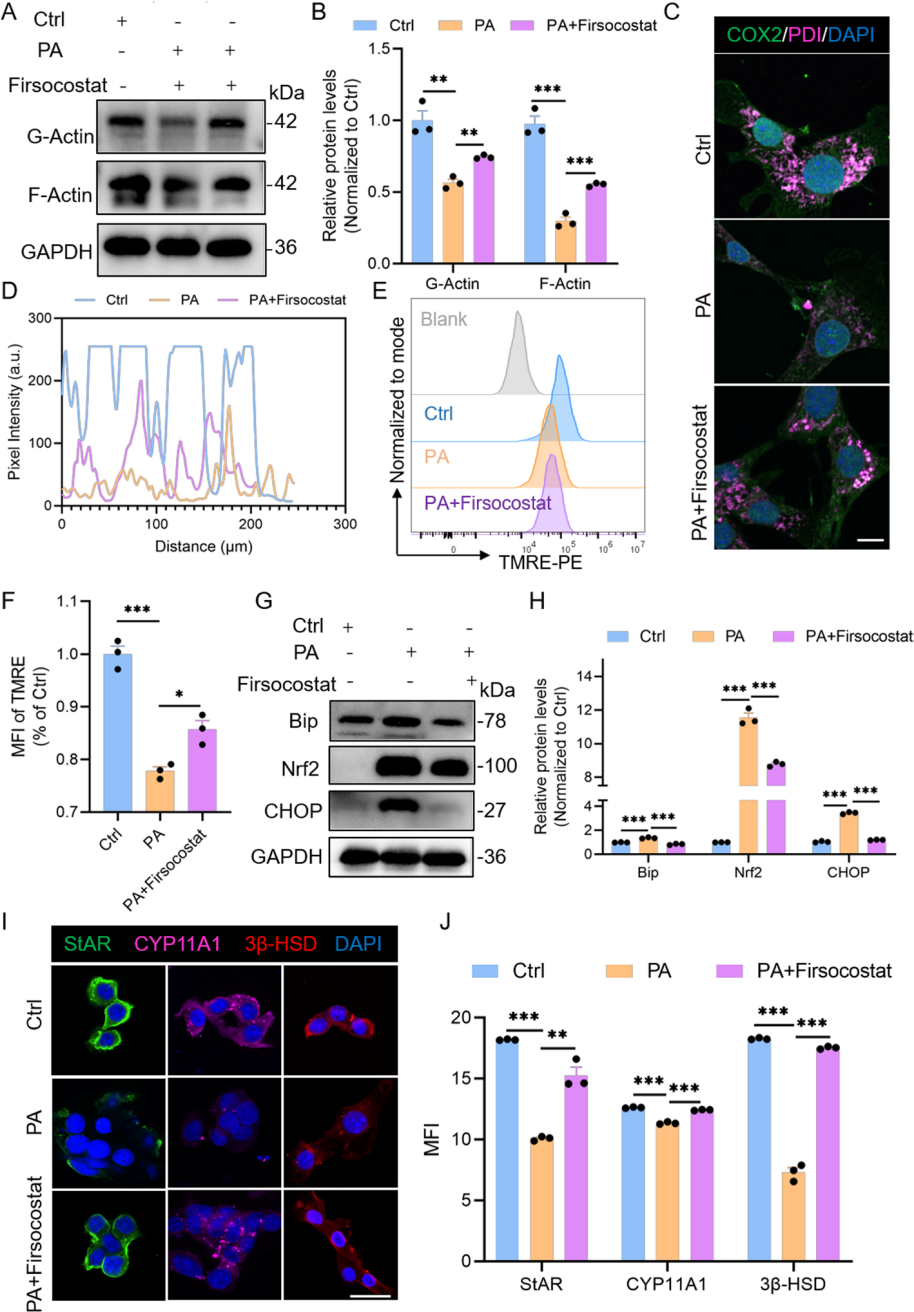
Promoted testosterone production via inhibiting LD synthesis, enhancing mitochondrial and ER performance in vitro. **A**, **B** WB analysis of G-actin and F-actin levels in TM3 cells across Ctrl, PA, and PA + Firsocostat treatment groups (*n* = 3 biological replicates per group). Firsocostat is an ACC inhibitor. **C** Representative immunofluorescence images showing COX2 (green) and PDI (magenta) in Ctrl, PA, and PA + Firsocostat treated TM3 cells. Scale bar: 10 μm. **D** Colocalization analysis of COX2 and PDI based on the images in (**C**). **E** Mitochondrial membrane potential was assessed in TM3 cells using a TMRE assay analyzed by flow cytometry. **F** Quantification of the mean TMRE fluorescence intensity from (**E**). **G**, **H** WB analysis of ER stress-related proteins in Ctrl, PA, and PA + Firsocostat-treated TM3 cells. **I** Representative immunofluorescence images of LC markers in Ctrl, PA, and PA + Firsocostat-treated TM3 cells. Scale bar: 25 μm. **J** Quantitative analysis of the mean fluorescence intensity of LC markers from (**I**). Data are presented as mean ± SEM (*n* = 3 biological replicates per group). Data were analyzed using multiple *t* test (**B**, **H**, **J**), one-way ANOVA (**F**). **p < 0.01; ***p < 0.001

Firsocostat is a known acetyl-CoA carboxylase (ACC) inhibitor that effectively suppresses intracellular fatty acid synthesis without notably affecting cell number [35]. To test whether it can mitigate the detrimental effects of lipid droplets on LCs, we administered Firsocostat in the HFD model. Firsocostat treatment markedly reversed the HFD-induced reductions in G-actin and F-actin expression (Fig. 5A, B). It also effectively rescued lipid-droplet-associated disruption of MERCs (Fig. 5C, D). To determine whether organelle function was restored, we assessed mitochondrial and ER parameters. Firsocostat significantly improved mitochondrial membrane potential in LCs (Fig. 5E, F). ER stress–related protein levels also trended toward normalization (Fig. 5G, H), indicating reestablishment of ER homeostasis. Finally, key markers of testosterone biosynthesis were evaluated. After Firsocostat treatment, expression of critical steroidogenic enzymes (Star, Cyp11a1, and 3β-HSD) increased significantly (Fig. 5I, J), demonstrating that restoration of mitochondrial and ER function promotes recovery of steroidogenic capacity.

### Firsocostat rescues testosterone biosynthesis in HFD mice

**Fig. 6:**
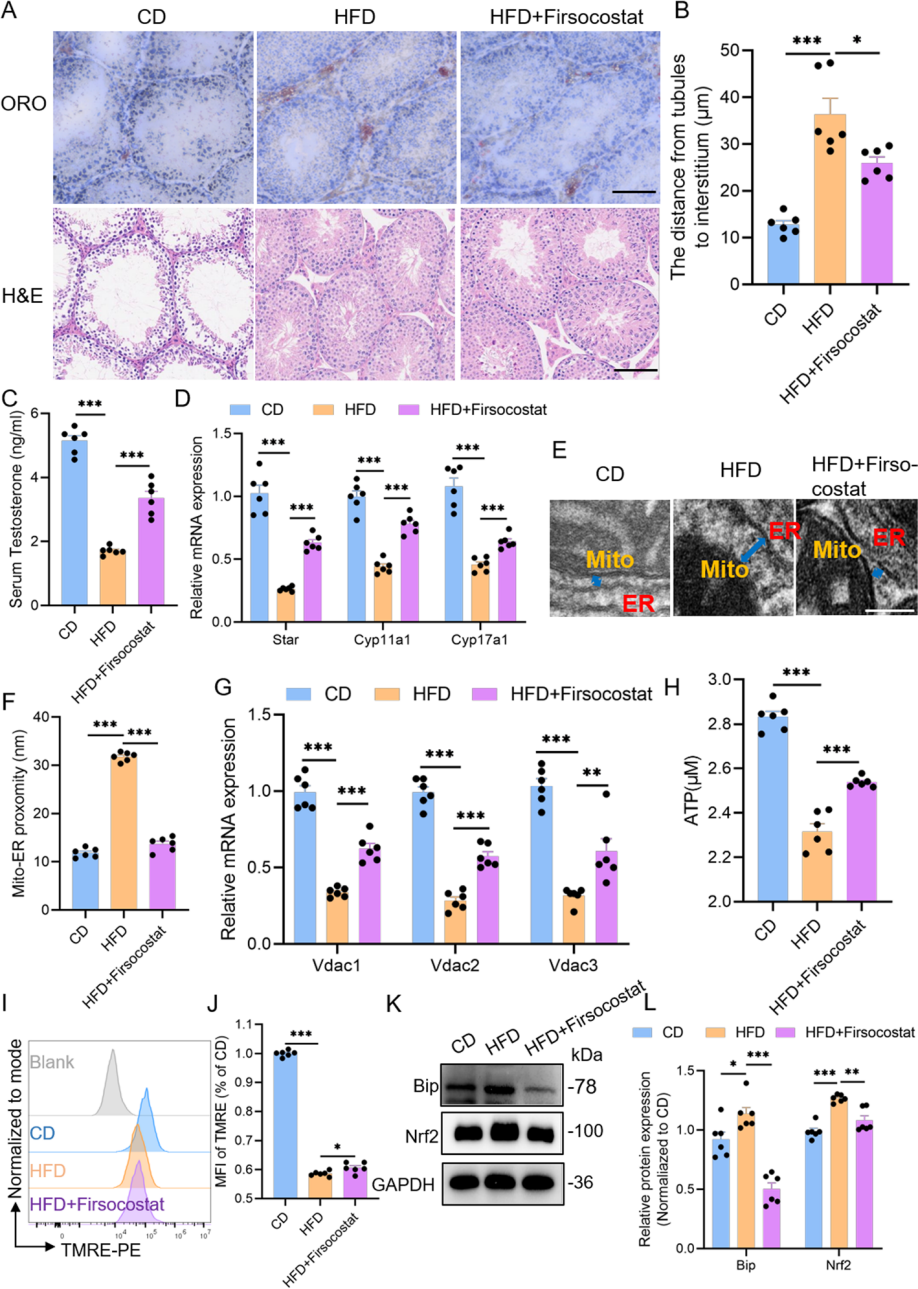
Inhibiting LD synthesis promotes MERCs and restores testosterone levels in vivo. **A** Representative testicular sections from CD, HFD, and HFD + Firsocostat mice, stained with ORO and H&E. Scale bars: 100 μm. **B** Quantification of the tubular-to-interstitium distance based on **A**. **C** Serum testosterone concentrations in CD, HFD, and HFD + Firsocostat mice. **D** Relative mRNA levels of LC markers in testicular tissue across the three groups. **E** TEM images showing LCs from CD, HFD, and HFD + Firsocostat testes. Scale bar: 100 nm. **F** Quantification of mitochondrial–ER contact sites (MERCs) from the images in (**E**) (*n* = 6 mitochondria from three fields per condition). **G** Relative mRNA expression of MERC-related genes in testicular tissue. **H** ATP content in LCs isolated from the testes of each group. **I** Mitochondrial membrane potential in LCs assessed by TMRE staining and flow cytometry. **J** Mean TMRE fluorescence intensity from (**I**). **K**, **L** WB analysis and quantification of ER stress-related proteins in CD, HFD and HFD + Firsocostat testes. Each dot represents a mouse. Data are presented as mean ± SEM. Data were analyzed using one-way ANOVA (**B**, **C**, **F**, **H**, **J**), Multiple *t* test (**D**, **L**). *p < 0.05; **p < 0.01; ***p < 0.001

Building on in vitro efficacy, we evaluated Firsocostat’s restorative effects on LC injury in HFD mice via intratesticular injection. Firsocostat markedly reduced lipid droplet accumulation in testes and significantly improved histopathology (Fig. 6A, B). Firsocostat also reduced HFD-induced testicular ROS elevation (Supplementary Fig. S4A, B), a finding corroborated by CellROX staining (Supplementary Fig. S4C, D). Serum testosterone measured by ELISA was significantly elevated after Firsocostat treatment (Fig. 6C), and transcription of key steroidogenic genes (Star, Cyp11a1, and Cyp17a1) was substantially restored (Fig. 6D). TEM showed that MERC architecture approached normal following treatment (Fig. 6E, F), accompanied by robust recovery of VDAC1, VDAC2, and VDAC3 protein levels (Fig. 6G), indicating amelioration of HFD-induced MERC disruption. Restoration of MERCs was associated with increased ATP production in LCs (Fig. 6H), reduced mitochondrial superoxide (Supplementary Fig. S4E, F), and recovery of mitochondrial membrane potential (Fig. 6I, J). ER stress markers also showed improving trends (Fig. 6K, L). To investigate whether Firsocostat could enhance testosterone synthesis in normal LC, we treated Ctrl TM3 cells with Firsocostat and found that it did not increase testosterone production (Supplementary Fig. S4G). Consistently, Firsocostat treatment in CD mice did not elevate serum testosterone levels (Supplementary Fig. S4H), confirming that Firsocostat does not stimulate testosterone synthesis under physiological conditions. In summary, Firsocostat restores testicular lipid homeostasis, alleviates HFD-induced MERC structural damage, promotes recovery of mitochondrial and ER function, and ultimately rescues testosterone biosynthesis in HFD mice.

## Discussion

The increasing prevalence of high-fat diet-induced metabolic disorders poses a significant threat to health [2–6]. Testosterone, a critical sex hormone in males, has an incompletely understood relationship with lipid metabolic dysregulation. In this study, we demonstrate that a high-fat environment substantially suppresses testosterone synthesis in LCs. Mechanistically, high-fat conditions promote intracellular lipid droplet accumulation, which disrupts actin-mediated mitochondria–endoplasmic reticulum contacts by altering cytoskeletal dynamics. This disruption leads to mitochondrial dysfunction and ER stress, ultimately downregulating key steroidogenic enzymes and reducing testosterone production. Our study elucidates a novel pathway—centered on the lipid droplet-cytoskeleton-organelle interaction network—underlying high-fat diet-associated testosterone deficiency. Furthermore, we show that administration of the specific ACC inhibitor Firsocostat suppresses de novo lipogenesis and lipid droplet formation, restores mitochondria–ER contact integrity and organelle function, enhances steroidogenic capacity in LCs, and consequently elevates testosterone levels, thereby ameliorating high-fat diet-induced reproductive impairment.

LCs serve as the primary functional units for androgen biosynthesis, with their steroidogenic process critically reliant on precise interorganellar coordination [11]. In the mitochondria of LCs, cholesterol from the bloodstream is converted to pregnenolone by CYP11A1 through side-chain cleavage, marking the first step in steroidogenesis [36]. After this conversion, pregnenolone is transported back to the cytosol and enters the endoplasmic reticulum. There, it undergoes a series of enzymatic reactions, including sequential catalysis by 3β-HSD and CYP17A1, ultimately leading to the production of testosterone [13, 37, 38]. This complex biosynthetic pathway spatially bridges mitochondria and the ER, underscoring the importance of efficient interorganellar communication for maintaining testosterone synthesis homeostasis. MERCs serve as critical communication platforms that not only facilitate rapid transfer of lipophilic intermediates such as cholesterol and pregnenolone via membrane apposition but also regulate Ca^2+^ signaling and redox homeostasis—processes essential for fine-tuning steroidogenic enzyme activities [31, 39, 40]. We provide evidence that, in a high-fat diet model, disruption of MERCs leads to mitochondrial dysfunction characterized by membrane potential depolarization and elevated reactive oxygen species, concurrent with ER stress marked by upregulation of the molecular chaperone Bip and the transcription factor CHOP. This dual dysregulation of mitochondrial and ER functionality collectively contributes to the suppression of testosterone biosynthesis.

Mitochondrial and endoplasmic reticulum function is influenced by numerous factors, including metabolic stress, oxidative stress, and calcium dyshomeostasis, with membrane contact sites between these organelles serving as a fundamental structural basis for their functional coordination [41]. Accumulating evidence indicates that mitochondria–ER contact sites function as direct communication bridges and act as central hubs regulating their synergistic activities. MERCs are dynamic interorganellar structures whose abundance and molecular composition adapt in response to cellular metabolic status [40, 42]. However, the dynamics and pathological significance of MERCs in LCs under high fat-related metabolic stress remain poorly understood. Through single-cell RNA sequencing analysis of high-fat diet mouse models, we demonstrated that LCs from HFD-fed mice exhibit significant downregulation of MERCs-related gene expression. This transcriptional alteration was further corroborated by transmission electron microscopy and immunofluorescence co-localization analyses, which confirmed a substantial reduction in close membrane apposition between mitochondria and the ER.

The cytoskeleton constitutes a three-dimensional protein filament network within eukaryotic cells, primarily composed of microfilaments, microtubules, and intermediate filaments [43–45]. Accumulating evidence demonstrates that cytoskeletal abnormalities are critically involved in the pathogenesis of numerous diseases. For instance, amyotrophic lateral sclerosis pathogenesis involves dysfunctional interactions between the cytoskeleton and mitochondria [46, 47]. Notably, microfilaments—formed by polymerization of actin monomers into filamentous actin with a pool of monomeric globular actin maintaining dynamic equilibrium—provide structural support for cell morphology, intracellular transport, and organelle anchoring. This dynamic equilibrium underlies the functional plasticity of the cytoskeleton, and its disruption has been implicated in various pathologies. In the central nervous system, disrupted actin dynamics in neurons lead to dendritic spine abnormalities, synaptic loss, and cognitive decline [48] ; in the renal filtration barrier, aberrant actin remodeling in podocytes causes foot process effacement, glomerular barrier disruption, and proteinuria [49] ; and in tumor cells, destabilized or dysregulated actin cytoskeleton facilitates migration, invasion, and metastasis [50]. Our study reveals that high-fat conditions significantly reduce both F-actin and G-actin expression levels in LCs, with immunofluorescence co-localization demonstrating disorganized spatial distribution of F-actin, disrupted actin-mediated mitochondria-ER close contacts, substantially diminished MERCs, and consequent mitochondrial dysfunction and ER stress.

Lipid droplets are dynamic organelles responsible for intracellular lipid storage. They primarily interact directly with other organelles through membrane contact sites, regulating intracellular lipid metabolism, energy homeostasis, and signal transduction [51, 52]. The accumulation of lipid droplets is a hallmark pathological feature of cellular lipotoxicity, with strong links to the pathogenesis of various metabolic conditions. For instance, excessive lipid droplets in the liver can disrupt mitochondrial and endoplasmic reticulum function [53, 54], and aberrant lipid droplets in pancreatic β-cells impairing insulin secretion [55]. Current endogenous lipid droplet clearance pathways present limitations: lipolysis is constrained by hormonal regulation, while lysosomal degradation and exocytotic mechanisms remain incompletely elucidated and difficult to modulate precisely. In contrast, inhibiting lipogenesis reduces lipid droplet biogenesis at its source. ACC, the key enzyme in lipogenesis, catalyzes the conversion of acetyl-CoA to malonyl-CoA—the essential precursor for fatty acid synthesis—and directly determines triacylglycerol production, making it a key therapeutic target [56]. The specific ACC inhibitor Firsocostat offers superior targeting compared to rapamycin or metformin by directly inhibiting ACC activity, reducing fatty acid synthesis substrates, decreasing triacylglycerol generation, while simultaneously protecting organelles from lipotoxic damage [57–59]. Our investigation demonstrated that Firsocostat treatment of LCs under high-fat conditions significantly reduced intracellular lipid droplet content, restored normal MERC architecture, upregulated key steroidogenic proteins, and markedly decreased reactive oxygen species levels. These findings indicate that lipid droplets are responsible for MERC disruption in LCs under high-fat conditions, and that Firsocostat ameliorates both lipid accumulation and functional impairment through suppression of de novo lipogenesis.

Numerous studies have established strong associations between various metabolic disorders and declining male reproductive function. For instance, hyperglycemia has been shown to induce testosterone secretion defects by promoting endoplasmic reticulum stress in LCs [60]. However, existing research predominantly focuses on dysfunction within individual organelles, lacking systematic investigation into inter-organellar crosstalk. Our study provides the first evidence that high-fat conditions disrupt LC function through a "lipid droplet-actin cytoskeleton-MERCs" axis, revealing that high-fat-induced lipid droplet accumulation regulates actin dynamics, thereby disrupting actin cytoskeleton-mediated mitochondria-ER contacts. Furthermore, we validate that the specific ACC inhibitor Firsocostat effectively suppresses de novo lipogenesis, restores MERCs and organelle function, and rescues testosterone synthesis and secretion capacity in LCs. Nevertheless, whether this "lipid droplet-actin cytoskeleton-MERCs" axis similarly affects other testicular cell types, including spermatogonia and Sertoli cells, requires further investigation. Collectively, our work innovatively links high-fat metabolic derangements to actin cytoskeleton reorganization and MERC disruption in LCs, expands the mechanistic understanding of high-fat diet effects on male reproduction, and provides both molecular targets and therapeutic strategies for intervening in high-fat-associated testosterone deficiency and male reproductive dysfunction.

Our work also has several limitations. First, we used the D12492 diet to establish an in vivo high-fat model, which is also commonly employed to develop obese mouse models [61–64]. Obesity encompasses phenotypes such as elevated blood glucose, insulin resistance, and weight gain. Although our study focuses more on the effects of lipids on the reproductive system, future research may need to consider systemic effects as well. Many dietary approaches have been documented for establishing an in vivo high-fat model [65–67]. A diet in which 60% of the energy comes from fat is typically defined as a HFD diet [68, 69]. The reason we chose the D12492 diet is that it is a widely reported high-fat diet, which demonstrates its reliability and reproducibility [64, 67, 70]. Second, our in vitro high-fat model was constructed using the Leydig cell line TM3, which is derived from Balb/c mice that are less prone to high fat model. While many studies have utilized the TM3 cell line to establish high-fat models [17, 71] and reported only minor differences between TM3 and primary LCs in terms of testosterone synthesis capacity and mitochondrial dynamics [20]—supporting its applicability—future in vitro studies could employ primary LCs or the MLTC-1 cell line derived from C57BL/6 mice to better reflect in vivo conditions. Last but not least, we used PA alone to create a high-fat model and investigate lipotoxicity mediated by LDs in LCs, omitting OA which is the most abundant fatty acid in the D12492 diet. In vitro high-fat models typically involve PA alone [72–74] or a combination of PA and OA[24, 75, 76] in specific ratios. However, literature suggests that OA exhibits a protective effect against apoptosis and lipotoxicity in vitro [77, 78], which represents a key distinction from PA. Therefore, given our research objectives, we opted to use PA alone. Subsequent studies could compare different modeling approaches in LCs to better mimic in vivo conditions.

## Conclusions

Our work establishes LDs as a leverage point for therapeutically restoring interorganelle connectivity. By targeting LD formation, we provide a conceptually new strategy—shifting from organelle-specific rescue to interorganelle communication repair—for treating high-fat diet-induced reproductive dysfunction.

## Supplementary Information

Additional file 1.

Additional file 2.

## Data Availability

The sc-RNA sequencing data are openly available in GEO at GSE239391.

## Abbreviations

HFD: High-fat diet
LC: Leydig cells
E: Endoplasmic reticulum
ROS: Reactive oxygen species
LDs: Lipid droplets
ACC: Acetyl-CoA carboxylase
MAMs: Mitochondria-associated membranes
FACS: Fluorescence-activated cell sorting
PA: Palmitic acid
AUC: Area under the curve
CASA: Computer-aided semen analysis
PFA: Paraformaldehyde
GEO: Gene Expression Omnibus
TEM: Transmission electron microscopy
H&E: Hematoxylin and eosin
SYCP3: Synaptonemal complex protein 3
CREM: cAMP responsive element modulator
ORO: Oil Red O
GSEA: Gene set enrichment analysis
RLU: Relative light units
DHE: Dihydroethidium
OD: Optical density
ANOVA: Analysis of variance
CD: Chow diet
MERCs: Mitochondria–ER contacts
SHBG: Sex hormone-binding globulin
Wdr1: WD repeat domain 1
Gsn: Gelsolin
Cfl1: Cofilin 1, non-muscle
Cfl2: Cofilin 2, muscle
Dstn: Destrin
Actr2: Actin-related protein 2
Actr3: ARP3 actin-related protein 3
Arpc3: Actin-related protein 2/3 complex, subunit 3
Arpc2: Actin-related protein 2/3 complex, subunit 2
Arpc1b: Actin-related protein 2/3 complex, subunit 1B
Arpc5: Actin-related protein 2/3 complex, subunit 5
Pip4k2a: Phosphatidylinositol-5-phosphate 4-kinase, type II, alpha
Braf B-Raf: Proto-oncogene, serine/threonine kinase
Akt2: Akt serine/threonine kinase 2
Actg1: Actin, gamma, cytoplasmic 1
Myh10: Myosin, heavy polypeptide 10, non-muscle
Myh11: Myosin, heavy polypeptide 11, smooth muscle
Pak3: P21 (RAC1) activated kinase 3
Vav2: Vav guanine nucleotide exchange factor 2
Pik3r1: Phosphoinositide-3-kinase regulatory subunit 1
Spata13: Spermatogenesis associated 13
Fgd3: FYVE, RhoGEF, and PH domain containing 3
Enah: ENAH actin regulator

## References

[1] Adolph TE, Tilg H. Western diets and chronic diseases. Nat Med. 2024;30(8):2133–47. 39085420 10.1038/s41591-024-03165-6

[2] Islam MA, Amin MN, Siddiqui SA, Hossain MP, Sultana F, Kabir MR. Trans fatty acids and lipid profile: a serious risk factor to cardiovascular disease, cancer and diabetes. Diabetes Metab Syndr. 2019;13(2):1643–7. 31336535 10.1016/j.dsx.2019.03.033

[3] Ortega FB, Lavie CJ, Blair SN. Obesity and cardiovascular disease. Circ Res. 2016;118(11):1752–70. 27230640 10.1161/CIRCRESAHA.115.306883

[4] Lian CY, Zhai ZZ, Li ZF, Wang L. High fat diet-triggered non-alcoholic fatty liver disease: a review of proposed mechanisms. Chem Biol Interact. 2020;330:109199. 32805210 10.1016/j.cbi.2020.109199

[5] Ren Q, Sun Q, Fu J. Dysfunction of autophagy in high-fat diet-induced non-alcoholic fatty liver disease. Autophagy. 2024;20(2):221–41. 37700498 10.1080/15548627.2023.2254191 PMC10813589

[6] Prasad M, Rajagopal P, Devarajan N, Veeraraghavan VP, Palanisamy CP, Cui B, et al. A comprehensive review on high -fat diet-induced diabetes mellitus: an epigenetic view. J Nutr Biochem. 2022;107:109037. 35533900 10.1016/j.jnutbio.2022.109037

[7] Zhang Z, Chen H, Li Q. High-fat diet led to testicular inflammation and ferroptosis via dysbiosis of gut microbes. Int Immunopharmacol. 2024;142(Pt B):113235. 39332089 10.1016/j.intimp.2024.113235

[8] Sanchez-Garrido MA, Ruiz-Pino F, Velasco I, Barroso A, Fernandois D, Heras V, et al. Intergenerational influence of paternal obesity on metabolic and reproductive health parameters of the offspring: male-preferential impact and involvement of Kiss1-mediated pathways. Endocrinology. 2018;159(2):1005–18. 29309558 10.1210/en.2017-00705

[9] Ding N, Zhang X, Zhang XD, Jing J, Liu SS, Mu YP, et al. Impairment of spermatogenesis and sperm motility by the high-fat diet-induced dysbiosis of gut microbes. Gut. 2020;69(9):1608–19. 31900292 10.1136/gutjnl-2019-319127 PMC7456731

[10] Senmaru T, Fukui M, Okada H, Mineoka Y, Yamazaki M, Tsujikawa M, et al. Testosterone deficiency induces markedly decreased serum triglycerides, increased small dense LDL, and hepatic steatosis mediated by dysregulation of lipid assembly and secretion in mice fed a high-fat diet. Metabolism. 2013;62(6):851–60. 23332447 10.1016/j.metabol.2012.12.007

[11] Zirkin BR, Papadopoulos V. Leydig cells: formation, function, and regulation. Biol Reprod. 2018;99(1):101–11. 29566165 10.1093/biolre/ioy059 PMC6044347

[12] Lei T, Yang Y, Yang WX. Luteinizing hormone regulates testosterone production, Leydig cell proliferation, differentiation, and circadian rhythm during spermatogenesis. Int J Mol Sci. 2025;26(8):3548. 40332028 10.3390/ijms26083548 PMC12027374

[13] Ye L, Su ZJ, Ge RS. Inhibitors of testosterone biosynthetic and metabolic activation enzymes. Molecules. 2011;16(12):9983–10001. 22138857 10.3390/molecules16129983 PMC6264586

[14] Ramalho-Santos J, Amaral S. Mitochondria and mammalian reproduction. Mol Cell Endocrinol. 2013;379(1–2):74–84. 23769709 10.1016/j.mce.2013.06.005

[15] Gao L, Gao D, Zhang J, Li C, Wu M, Xiao Y, et al. Age-related endoplasmic reticulum stress represses testosterone synthesis via attenuation of the circadian clock in Leydig cells. Theriogenology. 2022;189:137–49. 35753227 10.1016/j.theriogenology.2022.06.010

[16] Pinto-Fochi ME, Pytlowanciv EZ, Reame V, Rafacho A, Ribeiro DL, Taboga SR, et al. A high-fat diet fed during different periods of life impairs steroidogenesis of rat Leydig cells. Reproduction. 2016;152(6):795–808. 27679864 10.1530/REP-16-0072

[17] Wang J, Zhang S, Hu L, Wang Y, Liu K, Le J, et al. Pyrroloquinoline quinone inhibits PCSK9-NLRP3 mediated pyroptosis of Leydig cells in obese mice. Cell Death Dis. 2023;14(11):723. 37935689 10.1038/s41419-023-06162-8 PMC10630350

[18] Yu C, Jiang F, Zhang M, Luo D, Shao S, Zhao J, et al. HC diet inhibited testosterone synthesis by activating endoplasmic reticulum stress in testicular Leydig cells. J Cell Mol Med. 2019;23(5):3140–50. 30884106 10.1111/jcmm.14143 PMC6484377

[19] Wu L, Qu J, Mou L, Liu C. Apigenin improves testosterone synthesis by regulating endoplasmic reticulum stress. Biomed Pharmacother. 2024;177:117075. 38964181 10.1016/j.biopha.2024.117075

[20] Lv ZM, Liu C, Wang P, Chen YH. Dysregulation of mitochondrial dynamics and mitophagy are involved in high-fat diet-induced steroidogenesis inhibition. J Lipid Res. 2024;65(10):100639. 39236859 10.1016/j.jlr.2024.100639 PMC11467671

[21] Prinz WA, Toulmay A, Balla T. The functional universe of membrane contact sites. Nat Rev Mol Cell Biol. 2020;21(1):7–24. 31732717 10.1038/s41580-019-0180-9 PMC10619483

[22] Giacomello M, Pellegrini L. The coming of age of the mitochondria-ER contact: a matter of thickness. Cell Death Differ. 2016;23(9):1417–27. 27341186 10.1038/cdd.2016.52 PMC5072433

[23] Lee S, Min KT. The interface between ER and mitochondria: molecular compositions and functions. Mol Cells. 2018;41(12):1000–7. 30590907 10.14348/molcells.2018.0438 PMC6315321

[24] Zhang L, Zheng Y, Shao M, Chen A, Liu M, Sun W, et al. AlphaFold-based AI docking reveals AMPK/SIRT1-TFEB pathway modulation by traditional Chinese medicine in metabolic-associated fatty liver disease. Pharmacol Res. 2025;212:107617. 39832686 10.1016/j.phrs.2025.107617

[25] Boyman L, Karbowski M, Lederer WJ. Regulation of mitochondrial ATP production: Ca (^2+^) signaling and quality control. Trends Mol Med. 2020;26(1):21–39. 31767352 10.1016/j.molmed.2019.10.007 PMC7921598

[26] Szymanski J, Janikiewicz J, Michalska B, Patalas-Krawczyk P, Perrone M, Ziolkowski W, et al. Interaction of mitochondria with the endoplasmic reticulum and plasma membrane in calcium homeostasis, lipid trafficking and mitochondrial structure. Int J Mol Sci. 2017;18(7):1576. 28726733 10.3390/ijms18071576 PMC5536064

[27] Linden M, Nelson BD, Loncar D, Leterrier JF. Studies on the interaction between mitochondria and the cytoskeleton. J Bioenerg Biomembr. 1989;21(4):507–18. 2478536 10.1007/BF00762522

[28] Li S, Xu S, Roelofs BA, Boyman L, Lederer WJ, Sesaki H, et al. Transient assembly of F-actin on the outer mitochondrial membrane contributes to mitochondrial fission. J Cell Biol. 2015;208(1):109–23. 25547155 10.1083/jcb.201404050 PMC4284235

[29] Cao W, Zhang Y, Qi J, Zhang Y, Ding R, Meng B, et al. Single-cell transcriptome atlas of testes from mice with high-fat diets. Sci Data. 2024;11(1):573. 38834587 10.1038/s41597-024-03435-5 PMC11150238

[30] Luo P, Feng X, Deng R, Wang F, Zhang Y, Li X, et al. An autofluorescence-based isolation of Leydig cells for testosterone deficiency treatment. Mol Cell Endocrinol. 2021;535:111389. 34229003 10.1016/j.mce.2021.111389

[31] Yao S, Wei X, Deng W, Wang B, Cai J, Huang Y, et al. Nestin-dependent mitochondria-ER contacts define stem Leydig cell differentiation to attenuate male reproductive ageing. Nat Commun. 2022;13(1):4020. 35821241 10.1038/s41467-022-31755-w PMC9276759

[32] Mejhert N, Kuruvilla L, Gabriel KR, Elliott SD, Guie MA, Wang H, et al. Partitioning of MLX-family transcription factors to lipid droplets regulates metabolic gene expression. Mol Cell. 2020;77(6):1251-64 e9. 32023484 10.1016/j.molcel.2020.01.014 PMC7397554

[33] McLelland GL, Goiran T, Yi W, Dorval G, Chen CX, Lauinger ND, et al. Mfn2 ubiquitination by PINK1/parkin gates the p97-dependent release of ER from mitochondria to drive mitophagy. Elife. 2018;7:e32866. 29676259 10.7554/eLife.32866 PMC5927771

[34] Han L, Huang D, Wu S, Liu S, Wang C, Sheng Y, et al. Lipid droplet-associated lncRNA LIPTER preserves cardiac lipid metabolism. Nat Cell Biol. 2023;25(7):1033–46. 37264180 10.1038/s41556-023-01162-4 PMC10344779

[35] Harriman G, Greenwood J, Bhat S, Huang X, Wang R, Paul D, et al. Acetyl-CoA carboxylase inhibition by ND-630 reduces hepatic steatosis, improves insulin sensitivity, and modulates dyslipidemia in rats. Proc Natl Acad Sci USA. 2016;113(13):E1796–805. 26976583 10.1073/pnas.1520686113 PMC4822632

[36] Galano M, Li Y, Li L, Sottas C, Papadopoulos V. Role of constitutive STAR in Leydig cells. Int J Mol Sci. 2021. 10.3390/ijms22042021. 33670702 10.3390/ijms22042021 PMC7922663

[37] Preslocsk JP. Steroidogenesis in the mammalian testis. Endocr Rev. 1980;1(2):132–9. 7016514 10.1210/edrv-1-2-132

[38] Bose HS, Bose M, Whittal RM. Tom40 in cholesterol transport. iScience. 2023;26(4):106386. 37035007 10.1016/j.isci.2023.106386 PMC10074151

[39] Szabo L, Cummins N, Paganetti P, Odermatt A, Papassotiropoulos A, Karch C, et al. ER-mitochondria contacts and cholesterol metabolism are disrupted by disease-associated tau protein. EMBO Rep. 2023;24(8):e57499. 37401859 10.15252/embr.202357499 PMC10398652

[40] Goicoechea L, Conde de la Rosa L, Torres S, García-Ruiz C, Fernández-Checa JC. Mitochondrial cholesterol: Metabolism and impact on redox biology and disease. Redox Biol. 2023;61:102643. 10.1016/j.redox.2023.102643 PMC9989693 36857930

[41] Parlakgül G, Pang S, Artico LL, Min N, Cagampan E, Villa R, et al. Spatial mapping of hepatic ER and mitochondria architecture reveals zonated remodeling in fasting and obesity. Nat Commun. 2024;15(1):3982. 38729945 10.1038/s41467-024-48272-7 PMC11087507

[42] Ronayne CT, Latorre-Muro P. Navigating the landscape of mitochondrial-ER communication in health and disease. Front Mol Biosci. 2024;11:1356500. 38323074 10.3389/fmolb.2024.1356500 PMC10844478

[43] Fletcher DA, Mullins RD. Cell mechanics and the cytoskeleton. Nature. 2010;463(7280):485–92. 20110992 10.1038/nature08908 PMC2851742

[44] Kim S, Coulombe PA. Emerging role for the cytoskeleton as an organizer and regulator of translation. Nat Rev Mol Cell Biol. 2010;11(1):75–81. 20027187 10.1038/nrm2818

[45] Luna EJ, Hitt AL. Cytoskeleton–plasma membrane interactions. Science. 1992;258(5084):955–64. 1439807 10.1126/science.1439807

[46] Theunissen F, West PK, Brennan S, Petrović B, Hooshmand K, Akkari PA, et al. New perspectives on cytoskeletal dysregulation and mitochondrial mislocalization in amyotrophic lateral sclerosis. Transl Neurodegener. 2021;10(1):46. 34789332 10.1186/s40035-021-00272-z PMC8597313

[47] Lai WF, Wong WT. Roles of the actin cytoskeleton in aging and age-associated diseases. Ageing Res Rev. 2020;58:101021. 31968269 10.1016/j.arr.2020.101021

[48] Paciello F, Battistoni M, Martini S, Simone C, Pastore F, Sollazzo R, et al. Role of LIMK1-cofilin-actin axis in dendritic spine dynamics in Alzheimer’s disease. Cell Death Dis. 2025;16(1):431. 40461464 10.1038/s41419-025-07741-7 PMC12134335

[49] Qiu Y, Lei C, Zeng J, Xie Y, Cao Y, Yuan Q, et al. Asparagine endopeptidase protects podocytes in adriamycin-induced nephropathy by regulating actin dynamics through cleaving transgelin. Mol Ther. 2023;31(11):3337–54. 37689970 10.1016/j.ymthe.2023.09.003 PMC10638058

[50] Melica ME, Antonelli G, Semeraro R, La Regina G, Dafichi T, Fantini C, et al. Piezo1, F-Actin Remodeling, and Podocyte Survival and Regeneration. J Am Soc Nephrol. 2025;36(9):1749–63. 40172977 10.1681/ASN.0000000697 PMC12416952

[51] Olzmann JA, Carvalho P. Dynamics and functions of lipid droplets. Nat Rev Mol Cell Biol. 2019;20(3):137–55. 30523332 10.1038/s41580-018-0085-z PMC6746329

[52] Zadoorian A, Du X, Yang H. Lipid droplet biogenesis and functions in health and disease. Nat Rev Endocrinol. 2023;19(8):443–59. 37221402 10.1038/s41574-023-00845-0 PMC10204695

[53] Gluchowski NL, Becuwe M, Walther TC, Farese RV Jr. Lipid droplets and liver disease: from basic biology to clinical implications. Nat Rev Gastroenterol Hepatol. 2017;14(6):343–55. 28428634 10.1038/nrgastro.2017.32 PMC6319657

[54] Scorletti E, Carr RM. A new perspective on NAFLD: focusing on lipid droplets. J Hepatol. 2022;76(4):934–45. 34793866 10.1016/j.jhep.2021.11.009

[55] Tong X, Liu S, Stein R, Imai Y. Lipid droplets’ role in the regulation of β-cell function and β-cell demise in type 2 diabetes. Endocrinology. 2022. 10.1210/endocr/bqac007. 35086144 10.1210/endocr/bqac007 PMC8826878

[56] Mateo-Marín MA, Alves-Bezerra M. Targeting acetyl-CoA carboxylases for the treatment of MASLD. J Lipid Res. 2024;65(12):100676. 39461620 10.1016/j.jlr.2024.100676 PMC11621487

[57] Neokosmidis G, Cholongitas E, Tziomalos K. Acetyl-CoA carboxylase inhibitors in non-alcoholic steatohepatitis: is there a benefit? World J Gastroenterol. 2021;27(39):6522–6. 34754150 10.3748/wjg.v27.i39.6522 PMC8554398

[58] Younis IR, Nelson C, Weber EJ, Qin AR, Watkins TR, Othman AA. Pharmacokinetics and safety of Firsocostat, an Acetyl-Coenzyme A Carboxylase inhibitor, in participants with mild, moderate, and severe hepatic impairment. J Clin Pharmacol. 2024;64(7):878–86. 38520128 10.1002/jcph.2427

[59] Weber EJ, Younis IR, Nelson C, Qin AR, Watkins TR, Othman AA. Evaluation of the potential for Cytochrome P450 and transporter-mediated drug-drug interactions for Firsocostat, a liver-targeted inhibitor of Acetyl-CoA Carboxylase. Clin Pharmacokinet. 2024;63(10):1423–34. 39292376 10.1007/s40262-024-01420-0

[60] Wang D, Li Y, Zhai QQ, Zhu YF, Liu BY, Xu Y. Quercetin ameliorates testosterone secretion disorder by inhibiting endoplasmic reticulum stress through the miR-1306-5p/HSD17B7 axis in diabetic rats. Bosn J Basic Med Sci. 2022;22(2):191–204. 34582743 10.17305/bjbms.2021.6299 PMC8977087

[61] Sheng X, Zhu X, Zhang Y, Cui G, Peng L, Lu X, et al. Rhein protects against obesity and related metabolic disorders through liver X receptor-mediated uncoupling protein 1 upregulation in brown adipose tissue. Int J Biol Sci. 2012;8(10):1375–84. 23139635 10.7150/ijbs.4575 PMC3492795

[62] Papadopoulos G, Legaki AI, Georgila K, Vorkas P, Giannousi E, Stamatakis G, et al. Integrated omics analysis for characterization of the contribution of high fructose corn syrup to non-alcoholic fatty liver disease in obesity. Metabolism. 2023;144:155552. 36996933 10.1016/j.metabol.2023.155552

[63] Liu Y, Liu J, Ren R, Xin Z, Luo Y, Chen Y, et al. Short-term and long-term high-fat diet promote metabolic disorder through reprogramming mRNA m (6)A in white adipose tissue by gut microbiota. Microbiome. 2025;13(1):75. 40091072 10.1186/s40168-025-02047-4 PMC11912683

[64] Makhlouf M, Souza DG, Kurian S, Bellaver B, Ellis H, Kuboki A, et al. Short-term consumption of highly processed diets varying in macronutrient content impair the sense of smell and brain metabolism in mice. Mol Metab. 2024;79:101837. 37977411 10.1016/j.molmet.2023.101837 PMC10724696

[65] Liu Y, Yang K, Jia Y, Shi J, Tong Z, Fang D, et al. Gut microbiome alterations in high-fat-diet-fed mice are associated with antibiotic tolerance. Nat Microbiol. 2021;6(7):874–84. 34017107 10.1038/s41564-021-00912-0

[66] Towers AE, Oelschlager ML, Juda MB, Jain S, Gainey SJ, Freund GG. HFD refeeding in mice after fasting impairs learning by activating caspase-1 in the brain. Metabolism. 2020;102:153989. 31697963 10.1016/j.metabol.2019.153989 PMC6906226

[67] Okamura T, Hamaguchi M, Hasegawa Y, Hashimoto Y, Majima S, Senmaru T, et al. Oral exposure to polystyrene microplastics of mice on a normal or high-fat diet and intestinal and metabolic outcomes. Environ Health Perspect. 2023;131(2):27006. 36821708 10.1289/EHP11072 PMC9945580

[68] Zhuang H, Yao X, Li H, Li Q, Yang C, Wang C, et al. Long-term high-fat diet consumption by mice throughout adulthood induces neurobehavioral alterations and hippocampal neuronal remodeling accompanied by augmented microglial lipid accumulation. Brain Behav Immun. 2022;100:155–71. 34848340 10.1016/j.bbi.2021.11.018

[69] Lee ES, Kwon MH, Kim HM, Woo HB, Ahn CM, Chung CH. Curcumin analog CUR5-8 ameliorates nonalcoholic fatty liver disease in mice with high-fat diet-induced obesity. Metabolism. 2020;103:154015. 31758951 10.1016/j.metabol.2019.154015

[70] Soto JE, Burnett CML, Ten Eyck P, Abel ED, Grobe JL. Comparison of the effects of high-fat diet on energy flux in mice using two multiplexed metabolic phenotyping systems. Obesity (Silver Spring). 2019;27(5):793–802. 30938081 10.1002/oby.22441 PMC6478533

[71] Pan M, Li J, Wang Y, Liu Z, Li L, Wang T. The downregulation of hormone-sensitive lipase and dysregulation of cholesterol receptors/transporter affect testicular lipid homeostasis and function in HFD-induced oligoasthenospermia mice. Mol Med. 2025;31(1):274. 40760016 10.1186/s10020-025-01327-x PMC12323199

[72] Wu S, Tang W, Liu L, Wei K, Tang Y, Ma J, et al. Obesity-induced downregulation of miR-192 exacerbates lipopolysaccharide-induced acute lung injury by promoting macrophage activation. Cell Mol Biol Lett. 2024;29(1):36. 38486141 10.1186/s11658-024-00558-w PMC10938800

[73] Shimabukuro M, Higa M, Zhou YT, Wang MY, Newgard CB, Unger RH. Lipoapoptosis in beta-cells of obese prediabetic fa/fa rats. Role of serine palmitoyltransferase overexpression. J Biol Chem. 1998;273(49):32487–90. 9829981 10.1074/jbc.273.49.32487

[74] Chen Z, Wen D, Wang F, Wang C, Yang L. Curcumin protects against palmitic acid-induced apoptosis via the inhibition of endoplasmic reticulum stress in testicular Leydig cells. Reprod Biol Endocrinol. 2019;17(1):71. 31472681 10.1186/s12958-019-0517-4 PMC6717632

[75] Sun Y, Wang J, Guo X, Zhu N, Niu L, Ding X, et al. Oleic acid and eicosapentaenoic acid reverse palmitic acid-induced insulin resistance in human HepG2 cells via the reactive oxygen species/JUN pathway. Genomics Proteomics Bioinformatics. 2021;19(5):754–71. 33631425 10.1016/j.gpb.2019.06.005 PMC9170756

[76] Dong J, Li M, Peng R, Zhang Y, Qiao Z, Sun N. ACACA reduces lipid accumulation through dual regulation of lipid metabolism and mitochondrial function via AMPK- PPARalpha- CPT1A axis. J Transl Med. 2024;22(1):196. 38395901 10.1186/s12967-024-04942-0 PMC10885411

[77] Ricchi M, Odoardi MR, Carulli L, Anzivino C, Ballestri S, Pinetti A, et al. Differential effect of oleic and palmitic acid on lipid accumulation and apoptosis in cultured hepatocytes. J Gastroenterol Hepatol. 2009;24(5):830–40. 19207680 10.1111/j.1440-1746.2008.05733.x

[78] Zhang Y, Xiang Z, Xu Y, Cheung LS, Wang X, Wang M, et al. Oleic acid restores the impaired antitumor immunity of gammadelta-T cells induced by palmitic acid. Signal Transduct Target Ther. 2025;10(1):209. 40603316 10.1038/s41392-025-02295-8 PMC12222472

