# Supplementary material for: High-fat diet leads to male reproductive dysfunction by disrupting lipid-droplet-mediated organelle crosstalk
Source: Cell Mol Biol Lett. 2026 Mar 6;31:52. doi: 10.1186/s11658-026-00891-2 (PMC13077933; doi:10.1186/s11658-026-00891-2)
Supplement: Supplementary file 1 — Additional file 1. [file 11658_2026_891_MOESM1_ESM.docx]

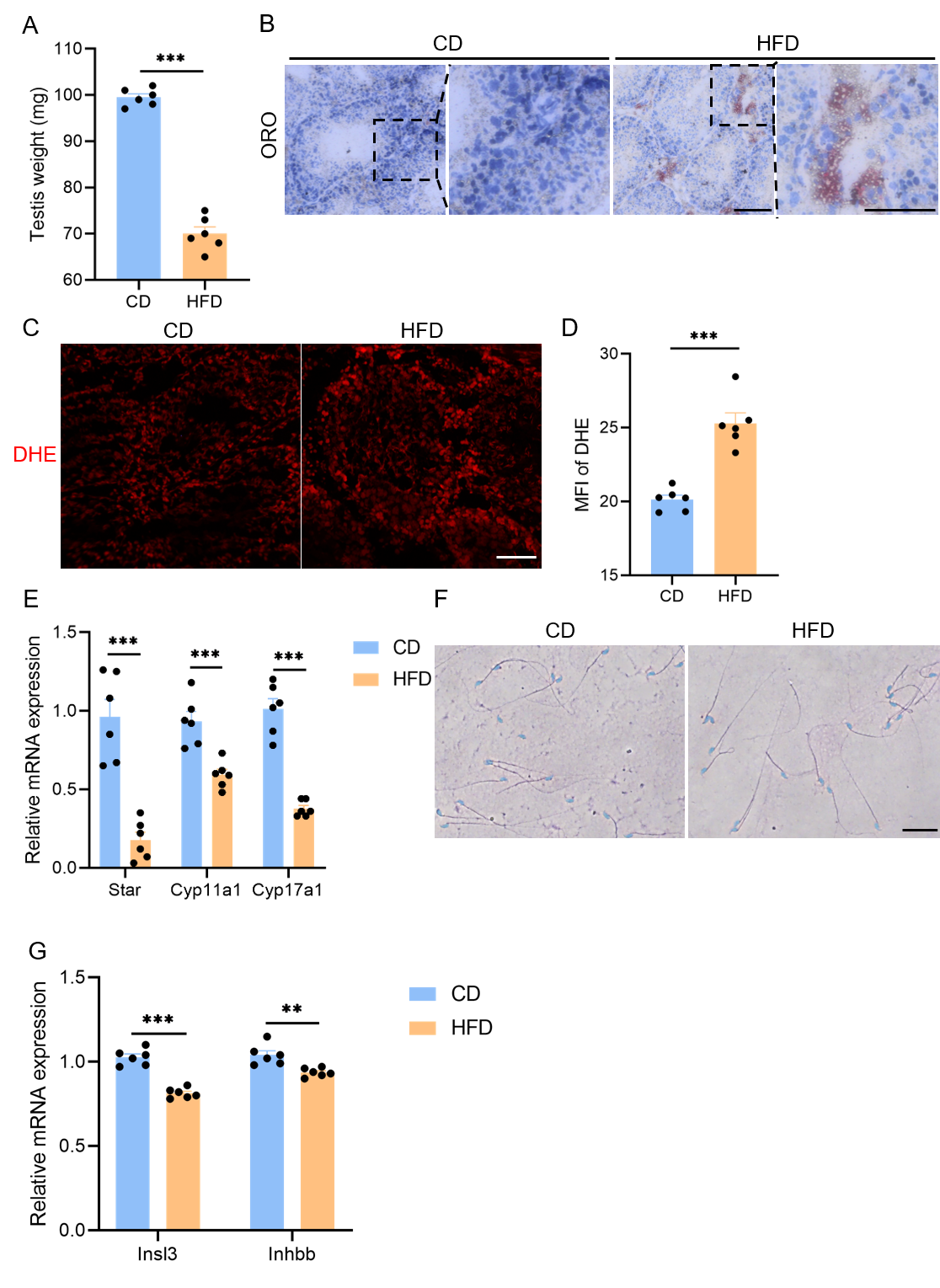


**Figure S1. Increased ROS levels and reduced testosterone synthesis ability under high-fat conditions.**

**(A)** Testis weight comparison between CD and HFD group. **(B)** Representative Oil Red O (ORO) staining pictures of CD and HFD testes. Scale bar: 100 μm for original pictures and 50 μm for enlarged pictures (n = 6 mice for each group). **(C)** Fluorescent staining of testis slices with DHE to detect ROS. Scale bar, 100 μm. **(D)** Quantitative analysis showing the MFI of DHE in **(C)** (n = 6 mice for each group). **(E)** qPCR analysis of relative mRNA expression of LC markers of testes from CD and HFD mice (n = 6 mice for each group). **(F)** Sperm Papanicolaou staining images of CD and HFD mice. Scale bar: 50 μm. **(G)** qPCR analysis of relative mRNA expression of Insl3 and Inhbb of testes from CD and HFD mice. Data are presented as mean±SEM. Data were analyzed using unpaired *t* test **(A, D)**, Multiple *t* test **(E, G)**. ***p* <0.01; ****p* <0.001.


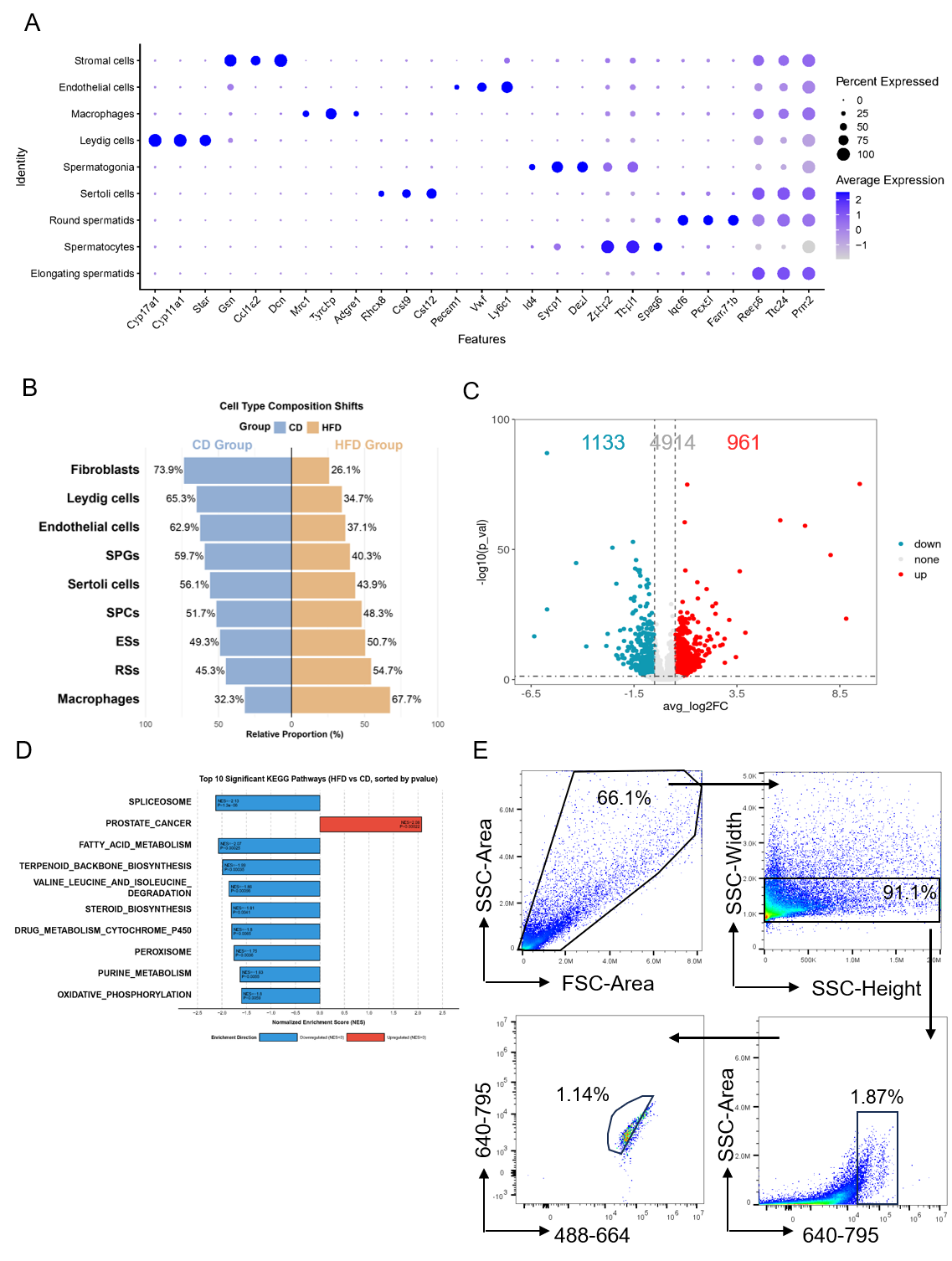


**Figure S2. LCs establish fewer MERCs under high-fat conditions.**

**(A)** Gene expression profiles of different cells in the testes of CD and HFD mice. **(B)** The cell ratio of different cells in CD and HFD testes. **(C)** The scatter plot of genes in CD and HFD testes. down: 1133 genes, none: 4914 genes, up: 961 genes. **(D)** The histogram showed the top 10 significant KEGG pathway in differential expressed genes of LCs in the CD and HFD testes. **(E)** Isolation of auto fluorescent LCs by FACS.


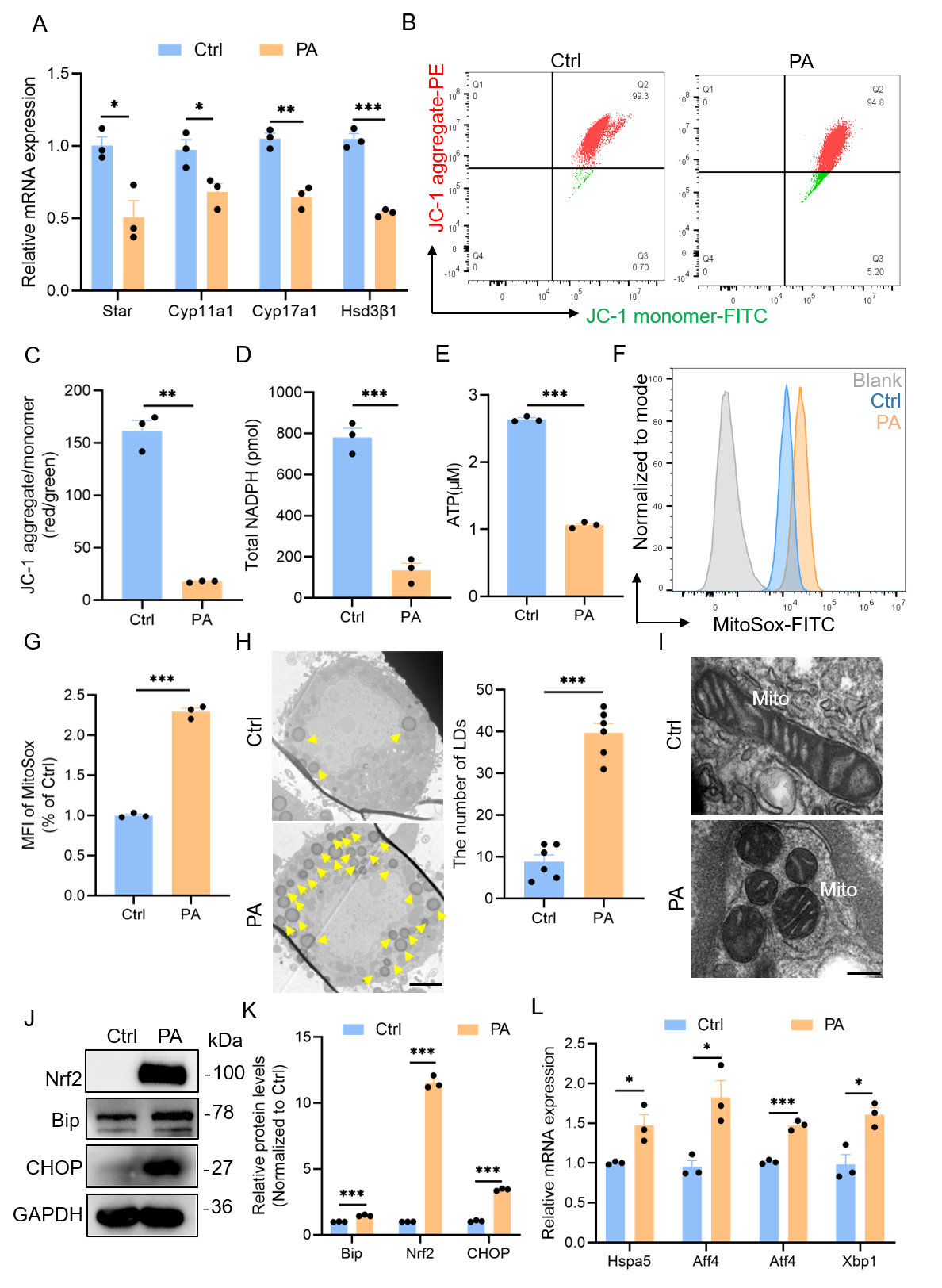


**Figure S3. Mitochondrial and ER dysfunction in LCs under high-fat conditions.**

**(A)** qPCR analysis of relative mRNA expression of LCs markers of testes from Ctrl and PA treated TM3 cells (n = 3 biological repeats for each group). **(B)** Mitochondrial membrane potential of TM3 cells was analyzed using JC-1 kit by flow cytometry. Q3: JC-1 monomer (green), Q2: JC-1 aggregate (red). **(C)** Quantification of the ratio of JC-1 aggregate and monomer from **(B)** (n=3 biological repeats for each group). **(D)** The total NADPH levels of Ctrl and PA treated TM3 cells (n=3 biological repeats for each group). **(E)** The levels of ATP in Ctrl and PA treated TM3 cells (n=3 biological repeats for each group). **(F)** Flow cytometry of mitochondrial ROS level stained with MitoSox of Ctrl and PA treated TM3 cells. **(G)** Quantification of mean fluorescence intensity of MitoSox in **(F)** (n=3 biological repeats for each group). **(H)** Representative TEM images of LDs indicated by yellow arrows in Ctrl and PA treated TM3 (left). Scale bar, 2 μm. Quantification of numbers of LDs (right). **(I)** Representative TEM images of TM3 mitochondria. Scale bar, 200 nm. **(J, K)** Western Blot analysis and quantification of ER stress-related proteins of Ctrl and PA treated TM3 cells (n=3 biological repeats for each group). **(L)** qPCR analysis of relative mRNA expression of ER stress-related genes of Ctrl and PA treated TM3 cells (n=3 biological repeats for each group). Data are presented as mean ± SEM. Data were analyzed using unpaired *t* test **(C-E, G, H)**, Multiple *t* test **(A, K, L)**. **p* <0.05; ***p* <0.01; ****p* <0. 001.


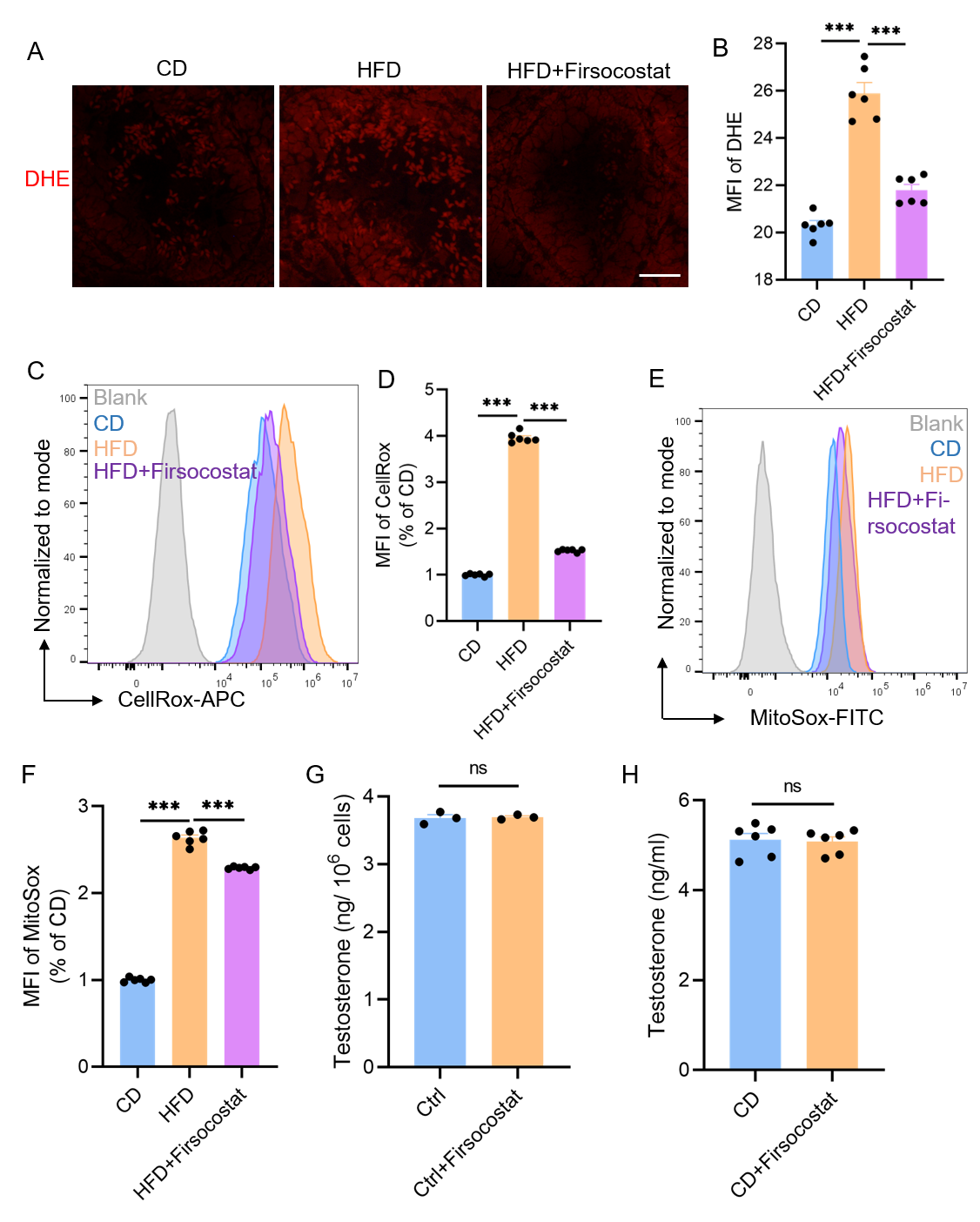


**Figure S4. Inhibiting LD synthesis reduces the oxidative stress levels**

**(A)** Fluorescent staining of testis slices with DHE to detect ROS. Scale bar, 100 μm. **(B)** Quantitative analysis showing the MFI of DHE in **(A)** (n = 6 mice for each group). **(C)** Flow cytometry of intracellular ROS level stained with CellRox of LCs from CD, HFD and HFD+Firsocostat testes. **(D)** Quantification of mean fluorescence intensity of CellRox in **(C)** (n=6 mice for each group). **(E)** Flow cytometry of mitochondrial ROS level stained with MitoSox of LCs from CD, HFD and HFD+Firsocostat testes. **(F)** Quantification of mean fluorescence intensity of MitoSox in **(E)** (n=6 mice for each group). **(G)**The levels of culture medium testosterone of Ctrl and Ctrl+Firsocostat TM3. **(H)** The levels of serum testosterone of CD and CD+Firsocostat mice. Data are presented as mean ± SEM. Data were analyzed using one-way ANOVA **(B, D, F)**, unpaired *t* test **(G, H)**. ****p* <0.001; ns, *p*≥0.05.
