# Supplementary material for: High-fat diet leads to male reproductive dysfunction by disrupting lipid-droplet-mediated organelle crosstalk
Source: Cell Mol Biol Lett. 2026 Mar 6;31:52. doi: 10.1186/s11658-026-00891-2 (PMC13077933; doi:10.1186/s11658-026-00891-2)
Supplement: Supplementary file 2 — Additional file 2. [file 11658_2026_891_MOESM2_ESM.pdf]

Repeat 1

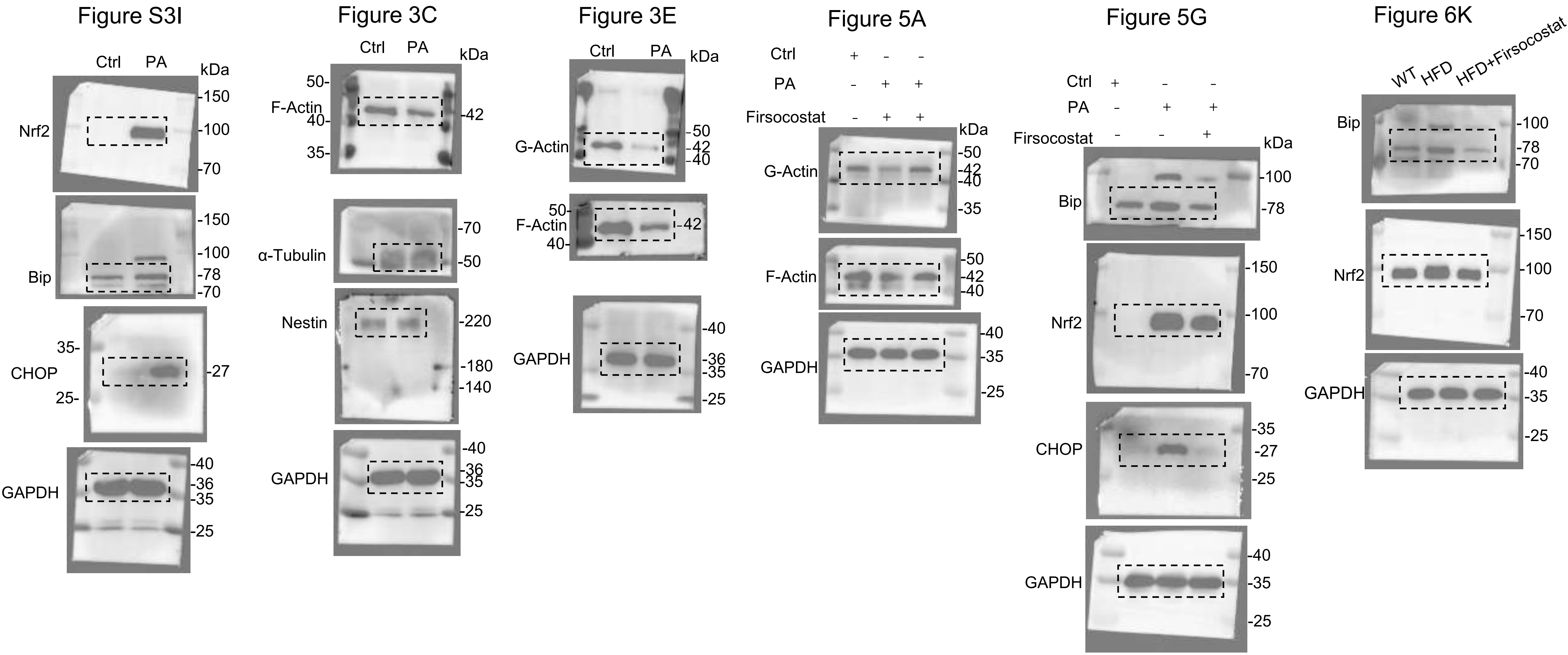

Figure S3I
Ctrl
PA
kDa
Nrf2
-150
-100
-70
Bip
-150
-100
-78
-70
CHOP
35-
25-
-27
GAPDH
-40
-36
-35
-25
Figure 3C
Ctrl
PA
kDa
F-Actin
50-
40-
35-
-42
α-Tubulin
-70
-50
Nestin
-220
-180
-140
GAPDH
-40
-36
-35
-25
Figure 3E
Ctrl
PA
kDa
G-Actin
-50
-42
-40
F-Actin
50-
40-
-42
GAPDH
-40
-36
-35
-25
Figure 5A
Ctrl + - -
PA - + +
Firsocostat - + +
kDa
G-Actin
-50
-42
-40
-35
F-Actin
-50
-42
-40
GAPDH
-40
-35
-25
Figure 5G
Ctrl + - -
PA - + +
Firsocostat - - +
kDa
Bip
-100
-78
Nrf2
-150
-100
-70
CHOP
-35
-27
-25
GAPDH
-40
-35
-25
Figure 6K
WT
HFD
HFD+Firsocostat
Bip
-100
-78
-70
Nrf2
-150
-100
-70
GAPDH
-40
-35
-25

Repeat 2

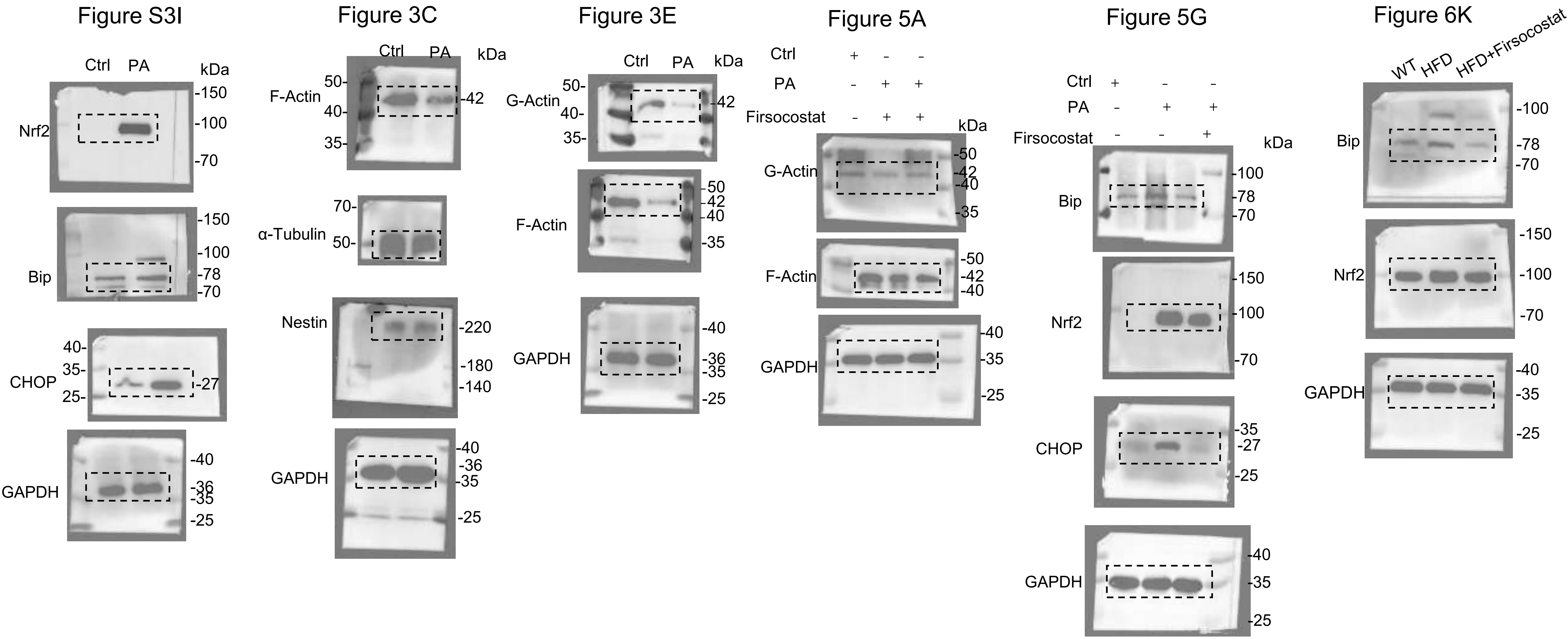

Figure S3I
Ctrl
PA
kDa
Nrf2
-150
-100
-70
Bip
-150
-100
-78
-70
CHOP
40-
35-
25-
-27
GAPDH
-40
-36
-35
-25
Figure 3C
Ctrl
PA
kDa
F-Actin
50-
40-
35-
-42
α-Tubulin
70-
50-
Nestin
-220
-180
-140
GAPDH
-40
-36
-35
-25
Figure 3E
Ctrl
PA
kDa
G-Actin
50-
40-
35-
-42
F-Actin
-50
-42
-40
-35
GAPDH
-40
-36
-35
-25
Figure 5A
Ctrl + - -
PA - + +
Firsocostat - + +
kDa
G-Actin
-50
-42
-40
-35
F-Actin
-50
-42
-40
GAPDH
-40
-35
-25
Figure 5G
Ctrl + - -
PA - + +
Firsocostat - - +
kDa
Bip
-100
-78
-70
Nrf2
-150
-100
-70
CHOP
-35
-27
-25
GAPDH
-40
-35
-25
Figure 6K
WT
HFD
HFD+Firsocostat
Bip
-100
-78
-70
Nrf2
-150
-100
-70
GAPDH
-40
-35
-25

Repeat 3

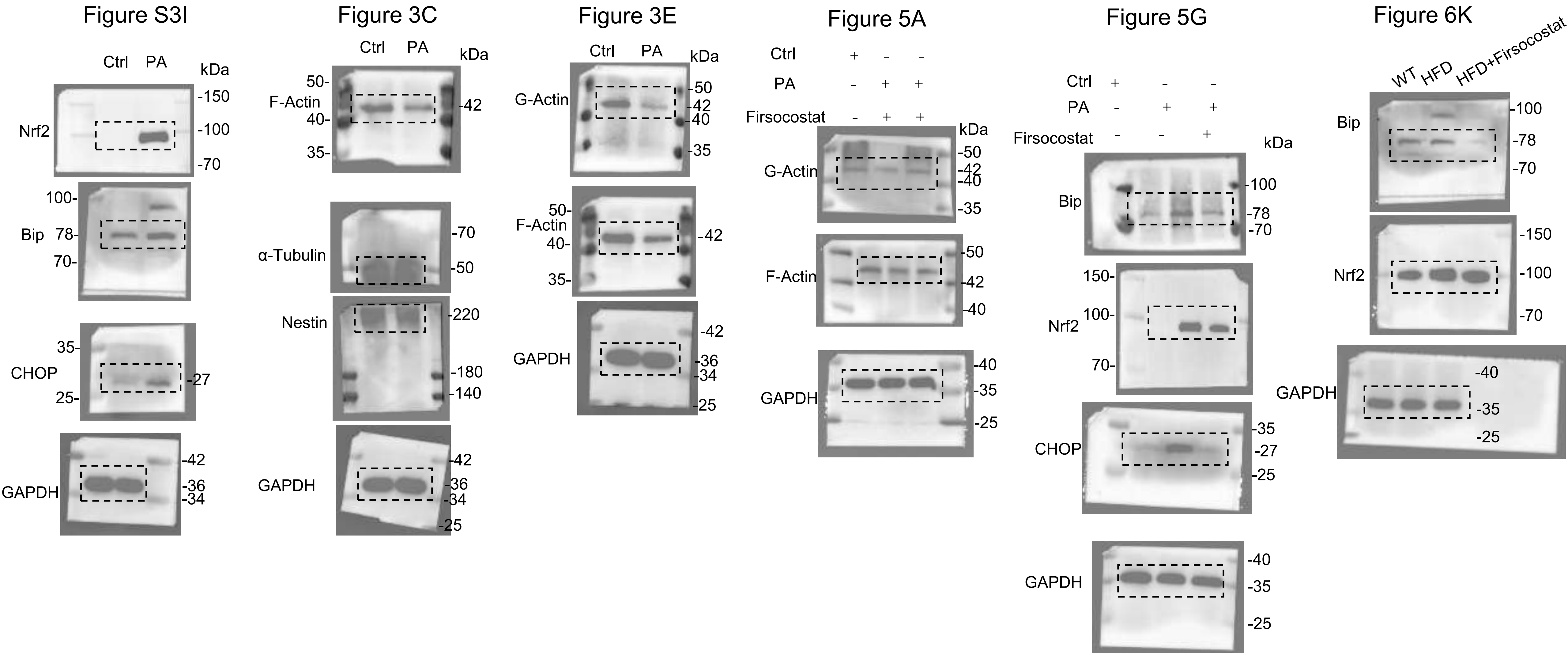
Figure S3I
Ctrl PA
kDa
Nrf2
-150
-100
-70
Bip
100-
78-
70-
CHOP
35-
-27
25-
GAPDH
-42
-36
-34
Figure 3C
Ctrl PA
kDa
F-Actin
50-
40-
35-
-42
α-Tubulin
-70
-50
Nestin
-220
-180
-140
GAPDH
-42
-36
-34
-25
Figure 3E
Ctrl PA
kDa
G-Actin
-50
-42
-40
-35
F-Actin
50-
40-
35-
-42
GAPDH
-42
-36
-34
-25
Figure 5A
Ctrl + - -
PA - + +
Firsocostat - + +
kDa
G-Actin
-50
-42
-40
-35
F-Actin
-50
-42
-40
GAPDH
-40
-35
-25
Figure 5G
Ctrl + - -
PA - + +
Firsocostat - - +
kDa
Bip
-100
-78
-70
Nrf2
150-
100-
70-
CHOP
-35
-27
-25
GAPDH
-40
-35
-25
Figure 6K
WT
HFD
HFD+Firsocostat
Bip
-100
-78
-70
Nrf2
-150
-100
-70
GAPDH
-40
-35
-25
